# Supplementary material for: Evolution of gene expression levels in the male reproductive organs of Anopheles mosquitoes
Source: Life Sci Alliance. 2019 Jan 2;2(1):e201800191. doi: 10.26508/lsa.201800191 (PMC6315087; doi:10.26508/lsa.201800191)
Supplement: Supplementary file 1 [file LSA-2018-00191_TableS1.pdf]

Genome	Tissue	Reference Assembly		Novel Assembly				
		**Exons**	**Introns**	**Exons**	**Introns**	**Covered by Ref**	**Covered by Novel**	**# Reads (million)**
An. gambiae s.s	MAGs	24 (95%)	55 (95%)	45 (94%)	120 (93%)	79 (29%)	165 (60%	225
	Testes	20 (79%)	42 (73%)	35 (73%)	87 (67%)	62 (23%)	121 (44%)	348
An. coluzzii	MAGs	19 (76%)	43 (74%)	29 (76%)	80 (75%)	62 (23%)	108 (40%)	51.4
	Testes	22 (91%)	50 (85%)	35 (91%)	93 (87%)	72 (26%)	128 (47%)	71.7
An. merus	MAGs	18 (74%)	24 (64%)	25 (77%)	55 (73%)	44 (15%)	81 (28%)	15.8
	Testes	19 (75%)	25 (61%)	25 (78%)	54 (71%)	44 (15%)	79 (27%)	9.46
An. arabiaensis	MAGs	18 (72%)	18 (66%)	25 (75%)	58 (75%)	36 (15%)	84 (34%)	15.7
	Testes	20 (83%)	20 (71%)	29 (85%)	65 (83%)	40 (16%)	93 (38%)	17.4
An. quadriannulatus	MAGs	19 (77%)	19 (71%)	27 (80%)	67 (79%)	38 (13%)	94 (32%)	23.6
	Testes	19 (78%)	18 (67%)	27 (80%)	65 (77%)	37 (13%)	92 (32%)	14.3
