# Supplementary material for: Evolution of gene expression levels in the male reproductive organs of Anopheles mosquitoes
Source: Life Sci Alliance. 2019 Jan 2;2(1):e201800191. doi: 10.26508/lsa.201800191 (PMC6315087; doi:10.26508/lsa.201800191)
Supplement: Supplementary file 2 [file LSA-2018-00191_TableS2.pdf]

Transcript	Cluster
AGAP001023-RF	1
AGAP001622-RA	1
AGAP001805-RA	1
AGAP002401-RA	1
AGAP002629-RA	1
AGAP005131-RA	1
AGAP005845-RA	1
AGAP007121-RA	1
AGAP009863-RA	1
AGAP010347-RA	1
AGAP010623-RA	1
AGAP011629-RA	1
AGAP011828-RA	1
AGAP012056-RA	1
AGAP028138-RA	1
AGAP000109-RA	1
AGAP000313-RA	1
AGAP000523-RA	1
AGAP000679-RA	1
AGAP000901-RA	1
AGAP000935-RA	1
AGAP000952-RA	1
AGAP000964-RA	1
AGAP001138-RA	1
AGAP001151-RA	1
AGAP001823-RA	1
AGAP001826-RA	1
AGAP001910-RA	1
AGAP002033-RA	1
AGAP002122-RA	1
AGAP002306-RA	1
AGAP002395-RA	1
AGAP002413-RD	1
AGAP002437-RA	1
AGAP002465-RA	1
AGAP002667-RA	1
AGAP002685-RA	1
AGAP002826-RA	1
AGAP002884-RA	1
AGAP003025-RC	1
AGAP003153-RD	1
AGAP003325-RA	1
AGAP003532-RA	1
AGAP003538-RA	1
AGAP003556-RA	1
AGAP003599-RA	1
AGAP003757-RA	1
AGAP003767-RA	1
AGAP003790-RD	1
AGAP004422-RA	1
AGAP004428-RA	1
AGAP004462-RA	1
AGAP004596-RA	1

AGAP004744-RA	1
AGAP004887-RA	1
AGAP004919-RA	1
AGAP004987-RA	1
AGAP005046-RB	1
AGAP005095-RA	1
AGAP005134-RA	1
AGAP005427-RA	1
AGAP005802-RA	1
AGAP005991-RA	1
AGAP006099-RA	1
AGAP006275-RA	1
AGAP006459-RA	1
AGAP006602-RA	1
AGAP006612-RA	1
AGAP006638-RA	1
AGAP006782-RA	1
AGAP006871-RA	1
AGAP006879-RA	1
AGAP006936-RA	1
AGAP007050-RA	1
AGAP007120-RA	1
AGAP007339-RA	1
AGAP007400-RA	1
AGAP007580-RB	1
AGAP007740-RA	1
AGAP007768-RA	1
AGAP007777-RA	1
AGAP007793-RA	1
AGAP007852-RB	1
AGAP007904-RA	1
AGAP007939-RA	1
AGAP007957-RB	1
AGAP008018-RA	1
AGAP008227-RA	1
AGAP008898-RA	1
AGAP008916-RA	1
AGAP008955-RA	1
AGAP009031-RA	1
AGAP009176-RA	1
AGAP009324-RA	1
AGAP009441-RB	1
AGAP009473-RA	1
AGAP009508-RA	1
AGAP009510-RA	1
AGAP009652-RA	1
AGAP009850-RA	1
AGAP009920-RA	1
AGAP009944-RA	1
AGAP009998-RA	1
AGAP010039-RA	1
AGAP010065-RA	1
AGAP010173-RA	1
AGAP010216-RA	1

AGAP010252-RA	1
AGAP010257-RA	1
AGAP010298-RA	1
AGAP010404-RB	1
AGAP010591-RA	1
AGAP010592-RA	1
AGAP010608-RA	1
AGAP010640-RA	1
AGAP010876-RA	1
AGAP010892-RA	1
AGAP010895-RA	1
AGAP010933-RA	1
AGAP011077-RA	1
AGAP011092-RA	1
AGAP011131-RA	1
AGAP011173-RA	1
AGAP011203-RA	1
AGAP011208-RA	1
AGAP011298-RA	1
AGAP011300-RA	1
AGAP011319-RA	1
AGAP011706-RA	1
AGAP011777-RA	1
AGAP011802-RA	1
AGAP011896-RA	1
AGAP012100-RA	1
AGAP012138-RA	1
AGAP012184-RA	1
AGAP012284-RA	1
AGAP012304-RA	1
AGAP013333-RA	1
AGAP013365-RA	1
AGAP013544-RA	1
AGAP009367-RA	2
AGAP012706-RA	2
AGAP009360-RA	2
AGAP009365-RA	2
AGAP009369-RA	2
AGAP009370-RA	2
AGAP009371-RA	2
AGAP013756-RA	2
AGAP001039-RB	2
AGAP001649-RA	2
AGAP002181-RC	2
AGAP002429-RA	2
AGAP006926-RA	2
AGAP008019-RA	2
AGAP008116-RA	2
AGAP008833-RA	2
AGAP009001-RA	2
AGAP009190-RA	2
AGAP009372-RA	2
AGAP009429-RA	2
AGAP012680-RA	2

AGAP013150-RA	2
AGAP013192-RA	2
AGAP013346-RA	2
AGAP000284-RA	2
AGAP000747-RB	2
AGAP001765-RA	2
AGAP002456-RB	2
AGAP002463-RA	2
AGAP005239-RA	2
AGAP008487-RA	2
AGAP009099-RA	2
AGAP009373-RA	2
AGAP001497-RB	2
AGAP005823-RA	2
AGAP006088-RA	2
AGAP006184-RA	2
AGAP006260-RD	2
AGAP000327-RA	2
AGAP000755-RA	2
AGAP001799-RA	2
AGAP006206-RB	2
AGAP008182-RA	2
AGAP011460-RA	2
AGAP009368-RA	2
AGAP007162-RB	2
AGAP012305-RA	2
AGAP006688-RA	2
AGAP001240-RA	3
AGAP003995-RA	3
AGAP004195-RA	3
AGAP004607-RB	3
AGAP006103-RC	3
AGAP008632-RA	3
AGAP009641-RA	3
AGAP011062-RA	3
AGAP011539-RA	3
AGAP012332-RA	3
AGAP028175-RA	3
AGAP028552-RA	3
AGAP000340-RA	3
AGAP001212-RB	3
AGAP001352-RA	3
AGAP001477-RA	3
AGAP001956-RA	3
AGAP002011-RA	3
AGAP002143-RA	3
AGAP002236-RA	3
AGAP002309-RA	3
AGAP002587-RA	3
AGAP002595-RA	3
AGAP003066-RA	3
AGAP003227-RA	3
AGAP003237-RA	3
AGAP003339-RA	3

AGAP003433-RA	3
AGAP003578-RA	3
AGAP003688-RA	3
AGAP003766-RA	3
AGAP004008-RA	3
AGAP004164-RD	3
AGAP004208-RA	3
AGAP004297-RA	3
AGAP004303-RA	3
AGAP004312-RC	3
AGAP004376-RA	3
AGAP004378-RA	3
AGAP004633-RA	3
AGAP004778-RA	3
AGAP005319-RA	3
AGAP005362-RA	3
AGAP005463-RA	3
AGAP005519-RA	3
AGAP005783-RA	3
AGAP005829-RA	3
AGAP006024-RA	3
AGAP006135-RA	3
AGAP006170-RA	3
AGAP006195-RA	3
AGAP006227-RA	3
AGAP006380-RA	3
AGAP006395-RA	3
AGAP006414-RA	3
AGAP006442-RA	3
AGAP006521-RA	3
AGAP006643-RA	3
AGAP006709-RA	3
AGAP006724-RA	3
AGAP006735-RA	3
AGAP006736-RA	3
AGAP006773-RA	3
AGAP006795-RA	3
AGAP006803-RA	3
AGAP006887-RA	3
AGAP006932-RA	3
AGAP006964-RA	3
AGAP007334-RA	3
AGAP007423-RA	3
AGAP007675-RA	3
AGAP007718-RA	3
AGAP007823-RA	3
AGAP008065-RA	3
AGAP008095-RA	3
AGAP008176-RA	3
AGAP008223-RA	3
AGAP008275-RA	3
AGAP008310-RA	3
AGAP008337-RA	3
AGAP008377-RA	3

AGAP008502-RA	3
AGAP008526-RA	3
AGAP008593-RA	3
AGAP008661-RA	3
AGAP008735-RA	3
AGAP008815-RA	3
AGAP009027-RA	3
AGAP009052-RA	3
AGAP009056-RA	3
AGAP009129-RA	3
AGAP009195-RA	3
AGAP009196-RA	3
AGAP009295-RA	3
AGAP009516-RA	3
AGAP009594-RA	3
AGAP009733-RA	3
AGAP009770-RA	3
AGAP009792-RA	3
AGAP009946-RA	3
AGAP009991-RA	3
AGAP010031-RA	3
AGAP010161-RA	3
AGAP010203-RA	3
AGAP010330-RA	3
AGAP010383-RA	3
AGAP010406-RA	3
AGAP010479-RA	3
AGAP010595-RA	3
AGAP010764-RA	3
AGAP010849-RA	3
AGAP010865-RA	3
AGAP010972-RA	3
AGAP010973-RA	3
AGAP011061-RA	3
AGAP011078-RA	3
AGAP011219-RA	3
AGAP011476-RA	3
AGAP011743-RA	3
AGAP011888-RA	3
AGAP011909-RA	3
AGAP012107-RA	3
AGAP012166-RA	3
AGAP012275-RA	3
AGAP012340-RA	3
AGAP012400-RA	3
AGAP012529-RA	3
AGAP013001-RA	3
AGAP013146-RC	3
AGAP013268-RA	3
AGAP013292-RA	3
AGAP013332-RA	3
AGAP013414-RA	3
AGAP013732-RA	3
AGAP028006-RA	3

AGAP028442-RA	3
AGAP028456-RA	3
AGAP028464-RA	3
AGAP028503-RA	3
AGAP028517-RA	3
AGAP028543-RA	3
AGAP028556-RA	3
AGAP028561-RA	3
AGAP028594-RA	3
AGAP000415-RA	3
AGAP000550-RA	3
AGAP000604-RA	3
AGAP000692-RA	3
AGAP000694-RA	3
AGAP000881-RA	3
AGAP001112-RA	3
AGAP001182-RA	3
AGAP001202-RA	3
AGAP001293-RA	3
AGAP001346-RA	3
AGAP001680-RA	3
AGAP002008-RA	3
AGAP002009-RA	3
AGAP002068-RA	3
AGAP002136-RA	3
AGAP002344-RA	3
AGAP002436-RA	3
AGAP002478-RA	3
AGAP002515-RA	3
AGAP002623-RA	3
AGAP002828-RA	3
AGAP003022-RA	3
AGAP003554-RA	3
AGAP003679-RA	3
AGAP003895-RA	3
AGAP004457-RA	3
AGAP004529-RA	3
AGAP004809-RA	3
AGAP005241-RA	3
AGAP005420-RA	3
AGAP005710-RA	3
AGAP006398-RA	3
AGAP006416-RA	3
AGAP006617-RA	3
AGAP006729-RA	3
AGAP007664-RA	3
AGAP007678-RA	3
AGAP007700-RA	3
AGAP007745-RA	3
AGAP008044-RA	3
AGAP008359-RA	3
AGAP008647-RA	3
AGAP009026-RB	3
AGAP009241-RA	3

AGAP010596-RA	3
AGAP010854-RA	3
AGAP011322-RA	3
AGAP011453-RA	3
AGAP001498-RA	3
AGAP001531-RA	3
AGAP001775-RA	3
AGAP002365-RA	3
AGAP003120-RA	3
AGAP003713-RA	3
AGAP003747-RA	3
AGAP008213-RA	3
AGAP008507-RA	3
AGAP011524-RA	3
AGAP001205-RA	3
AGAP007053-RA	3
AGAP028115-RA	3
AGAP002643-RA	3
AGAP003342-RA	3
AGAP028536-RA	3
AGAP001777-RA	3
AGAP009740-RA	3
AGAP002100-RA	3
AGAP013434-RA	3
AGAP012357-RA	3
AGAP000117-RA	3
AGAP006824-RA	3
AGAP008727-RF	4
AGAP011799-RA	4
AGAP013351-RA	4
AGAP003242-RA	4
AGAP003879-RA	4
AGAP004381-RA	4
AGAP008122-RA	4
AGAP008141-RA	4
AGAP008635-RA	4
AGAP009188-RA	4
AGAP010199-RA	4
AGAP010288-RA	4
AGAP011086-RA	4
AGAP011472-RA	4
AGAP011945-RA	4
AGAP012019-RD	4
AGAP012271-RA	4
AGAP012397-RA	4
AGAP012463-RA	4
AGAP012926-RA	4
AGAP013004-RA	4
AGAP013128-RA	4
AGAP013165-RB	4
AGAP013186-RA	4
AGAP013394-RA	4
AGAP013405-RA	4
AGAP013750-RA	4

AGAP028410-RA	4
AGAP028740-RA	4
AGAP000274-RA	4
AGAP000392-RA	4
AGAP000438-RA	4
AGAP000566-RB	4
AGAP000624-RA	4
AGAP001057-RA	4
AGAP001133-RA	4
AGAP001134-RA	4
AGAP001196-RA	4
AGAP001417-RB	4
AGAP001424-RA	4
AGAP001430-RA	4
AGAP001447-RA	4
AGAP002062-RA	4
AGAP002099-RA	4
AGAP002216-RA	4
AGAP002299-RA	4
AGAP002301-RA	4
AGAP002305-RA	4
AGAP002307-RA	4
AGAP002568-RA	4
AGAP002686-RA	4
AGAP002776-RA	4
AGAP003155-RA	4
AGAP003184-RA	4
AGAP003228-RA	4
AGAP003293-RA	4
AGAP003336-RA	4
AGAP003795-RA	4
AGAP003800-RA	4
AGAP003811-RA	4
AGAP003979-RA	4
AGAP004011-RA	4
AGAP004080-RA	4
AGAP004139-RA	4
AGAP004141-RA	4
AGAP004147-RA	4
AGAP004149-RA	4
AGAP004221-RA	4
AGAP004245-RA	4
AGAP004337-RA	4
AGAP004459-RB	4
AGAP004461-RB	4
AGAP004492-RA	4
AGAP004626-RA	4
AGAP004722-RA	4
AGAP004891-RA	4
AGAP004965-RB	4
AGAP005116-RA	4
AGAP005128-RA	4
AGAP005129-RA	4
AGAP005206-RA	4

AGAP005260-RA	4
AGAP005269-RA	4
AGAP005295-RA	4
AGAP005320-RA	4
AGAP005413-RA	4
AGAP005437-RA	4
AGAP005460-RA	4
AGAP005478-RA	4
AGAP005494-RA	4
AGAP005684-RA	4
AGAP005797-RA	4
AGAP005830-RA	4
AGAP005844-RA	4
AGAP005887-RA	4
AGAP005917-RA	4
AGAP006272-RA	4
AGAP006348-RA	4
AGAP006359-RA	4
AGAP006388-RA	4
AGAP006394-RA	4
AGAP006408-RA	4
AGAP006437-RA	4
AGAP006470-RA	4
AGAP006611-RA	4
AGAP006621-RA	4
AGAP006649-RA	4
AGAP006662-RA	4
AGAP006799-RA	4
AGAP006883-RA	4
AGAP006924-RA	4
AGAP006934-RA	4
AGAP007202-RA	4
AGAP007206-RA	4
AGAP007540-RA	4
AGAP007637-RA	4
AGAP007671-RA	4
AGAP007693-RA	4
AGAP007701-RA	4
AGAP007741-RA	4
AGAP007869-RA	4
AGAP007893-RA	4
AGAP007917-RA	4
AGAP007984-RA	4
AGAP008002-RA	4
AGAP008049-RA	4
AGAP008147-RA	4
AGAP008270-RA	4
AGAP008353-RA	4
AGAP008414-RA	4
AGAP008492-RA	4
AGAP008540-RA	4
AGAP008549-RA	4
AGAP008651-RA	4
AGAP008888-RA	4

AGAP008907-RA	4
AGAP008921-RA	4
AGAP008950-RA	4
AGAP008970-RA	4
AGAP009032-RA	4
AGAP009035-RA	4
AGAP009108-RA	4
AGAP009446-RA	4
AGAP009459-RA	4
AGAP009504-RA	4
AGAP009578-RA	4
AGAP009600-RA	4
AGAP009661-RA	4
AGAP009737-RA	4
AGAP009747-RA	4
AGAP010251-RA	4
AGAP010253-RA	4
AGAP010498-RA	4
AGAP010593-RA	4
AGAP011164-RA	4
AGAP011324-RA	4
AGAP011338-RA	4
AGAP011368-RA	4
AGAP011448-RA	4
AGAP011549-RA	4
AGAP011635-RA	4
AGAP011774-RA	4
AGAP011816-RA	4
AGAP011844-RA	4
AGAP011895-RA	4
AGAP011961-RA	4
AGAP012112-RA	4
AGAP012143-RA	4
AGAP012159-RA	4
AGAP012192-RA	4
AGAP012249-RA	4
AGAP012250-RA	4
AGAP012287-RA	4
AGAP012309-RA	4
AGAP013156-RA	4
AGAP013725-RA	4
AGAP028633-RA	4
AGAP000310-RA	4
AGAP000543-RA	4
AGAP000555-RA	4
AGAP000565-RA	4
AGAP000623-RA	4
AGAP000680-RA	4
AGAP000757-RA	4
AGAP000843-RA	4
AGAP000934-RA	4
AGAP000977-RA	4
AGAP001159-RA	4
AGAP001225-RA	4

AGAP001276-RA	4
AGAP001277-RA	4
AGAP001342-RA	4
AGAP001351-RA	4
AGAP001368-RB	4
AGAP001463-RA	4
AGAP001528-RA	4
AGAP001702-RA	4
AGAP001753-RA	4
AGAP001767-RA	4
AGAP001796-RA	4
AGAP001801-RA	4
AGAP002064-RA	4
AGAP002097-RA	4
AGAP002111-RA	4
AGAP002248-RA	4
AGAP002276-RA	4
AGAP002282-RA	4
AGAP002345-RA	4
AGAP002517-RA	4
AGAP002524-RA	4
AGAP002555-RA	4
AGAP003062-RA	4
AGAP003267-RA	4
AGAP003536-RA	4
AGAP003594-RA	4
AGAP003858-RA	4
AGAP004030-RA	4
AGAP004070-RA	4
AGAP004118-RA	4
AGAP004384-RA	4
AGAP004408-RA	4
AGAP004514-RA	4
AGAP004755-RA	4
AGAP005321-RA	4
AGAP005431-RA	4
AGAP005496-RA	4
AGAP005818-RA	4
AGAP005850-RA	4
AGAP005874-RA	4
AGAP006084-RA	4
AGAP006245-RA	4
AGAP006323-RA	4
AGAP006696-RA	4
AGAP007063-RA	4
AGAP007246-RA	4
AGAP007481-RA	4
AGAP007855-RA	4
AGAP008374-RA	4
AGAP008416-RA	4
AGAP008418-RA	4
AGAP008493-RA	4
AGAP008874-RA	4
AGAP009049-RA	4

AGAP009140-RA	4
AGAP009153-RA	4
AGAP009437-RA	4
AGAP009934-RA	4
AGAP010244-RA	4
AGAP010559-RA	4
AGAP011439-RA	4
AGAP011458-RA	4
AGAP012029-RA	4
AGAP012314-RA	4
AGAP013051-RA	4
AGAP013125-RA	4
AGAP028542-RA	4
AGAP000361-RA	4
AGAP001008-RA	4
AGAP001524-RA	4
AGAP001670-RA	4
AGAP001682-RA	4
AGAP002098-RA	4
AGAP003526-RA	4
AGAP003535-RA	4
AGAP004534-RA	4
AGAP004587-RA	4
AGAP004911-RA	4
AGAP005212-RA	4
AGAP005415-RA	4
AGAP005643-RA	4
AGAP006371-RA	4
AGAP007519-RA	4
AGAP008132-RA	4
AGAP010246-RA	4
AGAP010984-RA	4
AGAP011497-RA	4
AGAP013011-RA	4
AGAP000496-RA	4
AGAP000617-RA	4
AGAP000978-RA	4
AGAP001143-RA	4
AGAP002349-RA	4
AGAP002402-RA	4
AGAP002975-RA	4
AGAP004064-RB	4
AGAP004412-RA	4
AGAP005141-RA	4
AGAP007453-RA	4
AGAP007524-RA	4
AGAP009335-RA	4
AGAP011960-RA	4
AGAP003558-RA	4
AGAP007125-RA	4
AGAP010177-RA	4
AGAP004085-RA	4
AGAP004319-RA	4
AGAP006814-RA	4

AGAP008267-RA	4
AGAP010936-RA	4
AGAP001314-RA	4
AGAP006699-RA	4
AGAP009615-RA	4
AGAP010179-RA	4
AGAP011345-RA	4
AGAP000183-RA	4
AGAP000627-RC	4
AGAP002333-RA	4
AGAP007258-RA	4
AGAP010220-RA	4
AGAP008854-RA	4
AGAP011906-RA	4
AGAP006783-RA	4
AGAP007025-RA	4
AGAP006386-RA	4
AGAP012031-RA	4
AGAP012295-RA	5
AGAP010607-RA	5
AGAP010670-RA	5
AGAP011494-RA	5
AGAP012082-RA	5
AGAP012294-RA	5
AGAP000126-RA	5
AGAP000402-RA	5
AGAP000471-RB	5
AGAP000475-RB	5
AGAP000572-RA	5
AGAP001222-RA	5
AGAP001462-RA	5
AGAP001952-RA	5
AGAP002120-RA	5
AGAP002161-RB	5
AGAP002951-RA	5
AGAP002954-RA	5
AGAP003085-RA	5
AGAP003090-RB	5
AGAP003332-RA	5
AGAP003706-RA	5
AGAP003803-RA	5
AGAP003842-RA	5
AGAP003865-RA	5
AGAP004025-RA	5
AGAP004216-RA	5
AGAP004654-RA	5
AGAP004657-RB	5
AGAP004683-RA	5
AGAP004768-RB	5
AGAP004880-RC	5
AGAP005060-RA	5
AGAP005180-RA	5
AGAP005221-RA	5
AGAP005371-RA	5

AGAP005406-RA	5
AGAP005411-RB	5
AGAP005474-RA	5
AGAP005878-RB	5
AGAP005949-RB	5
AGAP006391-RA	5
AGAP006409-RA	5
AGAP006778-RA	5
AGAP006820-RA	5
AGAP006950-RA	5
AGAP006970-RA	5
AGAP007211-RA	5
AGAP007213-RA	5
AGAP007240-RA	5
AGAP007254-RA	5
AGAP007255-RA	5
AGAP007412-RA	5
AGAP007459-RA	5
AGAP007590-RA	5
AGAP007651-RA	5
AGAP007732-RB	5
AGAP007749-RA	5
AGAP007811-RA	5
AGAP007836-RB	5
AGAP007987-RA	5
AGAP008034-RA	5
AGAP008106-RA	5
AGAP008206-RA	5
AGAP008207-RA	5
AGAP008287-RB	5
AGAP008375-RA	5
AGAP008551-RA	5
AGAP008571-RA	5
AGAP008686-RA	5
AGAP008704-RA	5
AGAP009058-RA	5
AGAP009069-RA	5
AGAP009079-RA	5
AGAP009263-RA	5
AGAP009385-RB	5
AGAP009435-RA	5
AGAP009536-RA	5
AGAP009700-RA	5
AGAP009750-RA	5
AGAP009771-RA	5
AGAP009815-RA	5
AGAP009887-RA	5
AGAP009923-RA	5
AGAP009971-RA	5
AGAP010005-RA	5
AGAP010051-RA	5
AGAP010080-RA	5
AGAP010159-RA	5
AGAP010450-RA	5

AGAP010483-RA	5
AGAP010544-RA	5
AGAP010586-RA	5
AGAP010588-RA	5
AGAP010871-RA	5
AGAP010954-RA	5
AGAP011042-RA	5
AGAP011098-RA	5
AGAP011101-RA	5
AGAP011329-RA	5
AGAP011347-RA	5
AGAP011378-RB	5
AGAP011387-RA	5
AGAP011573-RA	5
AGAP011616-RA	5
AGAP011778-RA	5
AGAP011803-RA	5
AGAP011814-RA	5
AGAP011834-RA	5
AGAP011846-RA	5
AGAP011887-RA	5
AGAP011944-RA	5
AGAP011971-RA	5
AGAP012077-RA	5
AGAP012174-RA	5
AGAP012308-RA	5
AGAP012362-RA	5
AGAP012517-RA	5
AGAP012655-RA	5
AGAP013384-RA	5
AGAP013462-RA	5
AGAP013741-RA	5
AGAP028144-RA	5
AGAP028148-RA	5
AGAP028489-RA	5
AGAP028601-RA	5
AGAP000081-RA	5
AGAP000199-RB	5
AGAP000325-RB	5
AGAP000426-RC	5
AGAP000447-RA	5
AGAP000864-RB	5
AGAP000954-RC	5
AGAP001025-RA	5
AGAP001108-RA	5
AGAP001235-RB	5
AGAP001290-RA	5
AGAP001373-RA	5
AGAP001503-RA	5
AGAP001538-RA	5
AGAP001778-RA	5
AGAP001794-RA	5
AGAP001951-RA	5
AGAP001980-RB	5

AGAP001997-RA	5
AGAP002095-RD	5
AGAP002131-RA	5
AGAP002315-RA	5
AGAP002393-RA	5
AGAP002483-RA	5
AGAP002526-RA	5
AGAP002690-RB	5
AGAP003078-RB	5
AGAP003446-RA	5
AGAP003460-RA	5
AGAP003486-RA	5
AGAP003517-RB	5
AGAP003632-RA	5
AGAP003733-RB	5
AGAP003982-RB	5
AGAP004352-RB	5
AGAP004501-RA	5
AGAP004711-RA	5
AGAP004794-RA	5
AGAP004985-RA	5
AGAP005220-RA	5
AGAP005570-RA	5
AGAP005585-RC	5
AGAP005776-RA	5
AGAP005858-RA	5
AGAP006048-RA	5
AGAP006083-RB	5
AGAP006363-RA	5
AGAP006653-RA	5
AGAP006830-RA	5
AGAP007658-RA	5
AGAP007853-RA	5
AGAP008082-RC	5
AGAP008151-RA	5
AGAP008530-RA	5
AGAP008853-RA	5
AGAP009080-RA	5
AGAP009591-RA	5
AGAP009616-RA	5
AGAP010610-RA	5
AGAP010894-RA	5
AGAP012145-RA	5
AGAP012695-RA	5
AGAP012847-RA	5
AGAP013168-RA	5
AGAP013466-RA	5
AGAP000385-RD	5
AGAP001181-RA	5
AGAP001821-RA	5
AGAP001914-RA	5
AGAP001935-RA	5
AGAP001975-RA	5
AGAP002059-RB	5

AGAP002339-RA	5
AGAP002516-RA	5
AGAP002525-RA	5
AGAP002609-RB	5
AGAP003297-RA	5
AGAP003587-RB	5
AGAP004435-RA	5
AGAP004935-RA	5
AGAP007473-RA	5
AGAP007934-RB	5
AGAP008305-RC	5
AGAP008800-RA	5
AGAP010188-RA	5
AGAP010405-RB	5
AGAP010600-RA	5
AGAP011160-RA	5
AGAP012290-RA	5
AGAP012330-RA	5
AGAP013338-RA	5
AGAP000321-RA	5
AGAP000733-RA	5
AGAP000806-RA	5
AGAP001021-RB	5
AGAP001720-RA	5
AGAP002405-RA	5
AGAP003141-RB	5
AGAP003598-RA	5
AGAP004380-RA	5
AGAP004678-RA	5
AGAP005055-RA	5
AGAP005071-RB	5
AGAP006893-RA	5
AGAP009091-RB	5
AGAP010384-RA	5
AGAP010477-RB	5
AGAP010519-RE	5
AGAP010873-RA	5
AGAP011994-RA	5
AGAP012204-RA	5
AGAP012291-RA	5
AGAP012636-RA	5
AGAP028038-RA	5
AGAP000756-RA	5
AGAP001045-RA	5
AGAP001065-RH	5
AGAP003541-RC	5
AGAP003589-RA	5
AGAP005289-RA	5
AGAP005848-RA	5
AGAP006050-RA	5
AGAP008204-RA	5
AGAP010196-RA	5
AGAP005240-RA	5
AGAP005324-RA	5

AGAP007520-RA	5
AGAP009095-RB	5
AGAP010267-RA	5
AGAP011132-RA	5
AGAP000951-RC	5
AGAP008798-RA	5
AGAP009347-RA	5
AGAP010320-RA	5
AGAP001283-RA	5
AGAP001681-RA	5
AGAP004399-RB	5
AGAP006273-RA	5
AGAP007393-RC	5
AGAP004002-RA	5
AGAP007373-RB	5
AGAP009787-RA	5
AGAP028100-RA	5
AGAP001706-RA	5
AGAP003786-RA	5
AGAP008000-RA	5
AGAP009671-RA	5
AGAP007374-RA	6
AGAP008905-RA	6
AGAP009325-RA	6
AGAP010223-RA	6
AGAP000586-RA	6
AGAP001976-RA	6
AGAP002289-RA	6
AGAP002407-RA	6
AGAP002795-RA	6
AGAP003410-RA	6
AGAP004211-RA	6
AGAP004294-RA	6
AGAP004339-RA	6
AGAP004340-RA	6
AGAP004463-RA	6
AGAP004964-RA	6
AGAP005081-RA	6
AGAP005294-RB	6
AGAP005336-RA	6
AGAP005422-RA	6
AGAP005467-RB	6
AGAP006043-RA	6
AGAP006756-RA	6
AGAP006953-RA	6
AGAP007144-RA	6
AGAP007168-RA	6
AGAP007491-RA	6
AGAP007668-RA	6
AGAP007699-RC	6
AGAP007810-RA	6
AGAP008163-RA	6
AGAP008405-RA	6
AGAP009094-RA	6

AGAP009127-RA	6
AGAP009192-RA	6
AGAP009570-RA	6
AGAP009635-RA	6
AGAP009648-RA	6
AGAP009989-RA	6
AGAP010064-RA	6
AGAP010622-RA	6
AGAP010925-RA	6
AGAP010961-RA	6
AGAP011423-RA	6
AGAP011842-RA	6
AGAP011845-RA	6
AGAP011931-RA	6
AGAP012088-RA	6
AGAP012195-RA	6
AGAP012420-RA	6
AGAP012642-RA	6
AGAP028194-RA	6
AGAP028485-RA	6
AGAP000082-RA	6
AGAP000112-RA	6
AGAP000414-RA	6
AGAP000598-RA	6
AGAP000603-RA	6
AGAP000807-RA	6
AGAP000851-RA	6
AGAP000923-RA	6
AGAP001165-RA	6
AGAP001285-RA	6
AGAP001387-RA	6
AGAP001414-RA	6
AGAP001526-RA	6
AGAP001620-RA	6
AGAP001650-RA	6
AGAP001711-RA	6
AGAP001712-RA	6
AGAP001732-RA	6
AGAP001804-RA	6
AGAP001819-RA	6
AGAP002137-RA	6
AGAP002183-RA	6
AGAP002389-RA	6
AGAP002754-RA	6
AGAP002921-RB	6
AGAP003136-RA	6
AGAP003217-RA	6
AGAP003621-RA	6
AGAP003819-RA	6
AGAP004400-RB	6
AGAP004668-RA	6
AGAP004818-RA	6
AGAP004849-RA	6
AGAP005149-RA	6

AGAP005459-RA	6
AGAP005649-RA	6
AGAP005685-RA	6
AGAP005814-RA	6
AGAP006684-RA	6
AGAP007146-RA	6
AGAP007781-RA	6
AGAP009491-RA	6
AGAP011829-RA	6
AGAP011946-RA	6
AGAP012587-RA	6
AGAP001217-RA	6
AGAP001502-RA	6
AGAP003089-RA	6
AGAP003134-RA	6
AGAP004330-RA	6
AGAP008254-RA	6
AGAP010606-RA	6
AGAP012361-RA	6
AGAP002764-RA	6
AGAP002962-RA	6
AGAP003411-RA	6
AGAP003770-RA	6
AGAP007363-RA	6
AGAP009106-RA	6
AGAP011301-RA	6
AGAP000541-RB	6
AGAP002412-RA	6
AGAP003462-RA	6
AGAP003477-RA	6
AGAP006173-RA	6
AGAP006802-RA	6
AGAP007966-RC	6
AGAP011548-RA	6
AGAP000444-RA	6
AGAP003919-RA	6
AGAP011080-RA	6
AGAP002247-RA	6
AGAP008339-RA	6
AGAP008773-RA	6
AGAP007312-RC	6
AGAP002308-RA	6
AGAP011364-RA	6
AGAP009200-RA	7
AGAP009958-RA	7
AGAP010171-RB	7
AGAP010866-RA	7
AGAP011174-RA	7
AGAP011690-RA	7
AGAP011717-RA	7
AGAP012093-RA	7
AGAP012183-RA	7
AGAP012186-RA	7
AGAP012350-RA	7

AGAP028514-RA	7
AGAP028599-RA	7
AGAP000241-RA	7
AGAP000776-RA	7
AGAP000781-RA	7
AGAP000815-RA	7
AGAP001380-RA	7
AGAP001381-RA	7
AGAP001399-RA	7
AGAP001407-RA	7
AGAP001476-RA	7
AGAP001507-RA	7
AGAP001613-RA	7
AGAP001810-RA	7
AGAP002016-RA	7
AGAP002028-RA	7
AGAP002167-RB	7
AGAP002351-RA	7
AGAP002415-RA	7
AGAP002418-RA	7
AGAP002419-RA	7
AGAP002481-RA	7
AGAP002482-RA	7
AGAP002518-RA	7
AGAP002704-RA	7
AGAP002718-RA	7
AGAP002896-RA	7
AGAP003000-RA	7
AGAP003030-RA	7
AGAP003052-RA	7
AGAP003095-RA	7
AGAP003099-RA	7
AGAP003135-RA	7
AGAP003177-RA	7
AGAP003206-RB	7
AGAP003514-RA	7
AGAP003608-RA	7
AGAP003612-RA	7
AGAP003619-RA	7
AGAP003878-RA	7
AGAP004031-RA	7
AGAP004092-RA	7
AGAP004373-RC	7
AGAP004395-RA	7
AGAP004625-RB	7
AGAP004631-RA	7
AGAP004929-RA	7
AGAP004937-RA	7
AGAP004977-RA	7
AGAP004988-RA	7
AGAP005293-RB	7
AGAP005393-RA	7
AGAP005549-RA	7
AGAP005576-RA	7

AGAP005752-RA	7
AGAP005934-RA	7
AGAP005996-RA	7
AGAP006246-RA	7
AGAP006339-RA	7
AGAP006373-RA	7
AGAP006518-RA	7
AGAP006546-RA	7
AGAP006670-RA	7
AGAP006718-RA	7
AGAP006809-RA	7
AGAP006937-RC	7
AGAP006942-RA	7
AGAP006963-RA	7
AGAP007119-RA	7
AGAP007175-RA	7
AGAP007201-RA	7
AGAP007302-RA	7
AGAP007418-RA	7
AGAP007478-RA	7
AGAP007532-RA	7
AGAP007596-RA	7
AGAP007653-RA	7
AGAP007654-RA	7
AGAP007667-RA	7
AGAP007713-RD	7
AGAP007885-RA	7
AGAP007919-RA	7
AGAP007926-RA	7
AGAP007954-RA	7
AGAP008086-RA	7
AGAP008297-RA	7
AGAP008369-RA	7
AGAP008376-RA	7
AGAP008419-RD	7
AGAP008536-RA	7
AGAP008617-RA	7
AGAP008633-RA	7
AGAP008729-RA	7
AGAP008749-RA	7
AGAP008785-RA	7
AGAP008843-RB	7
AGAP008895-RA	7
AGAP008917-RA	7
AGAP008918-RA	7
AGAP008952-RA	7
AGAP009104-RA	7
AGAP009135-RA	7
AGAP009172-RA	7
AGAP009181-RA	7
AGAP009202-RA	7
AGAP009224-RF	7
AGAP009237-RA	7
AGAP009310-RA	7

AGAP009463-RA	7
AGAP009505-RA	7
AGAP009523-RA	7
AGAP009569-RA	7
AGAP009624-RA	7
AGAP009852-RA	7
AGAP009871-RA	7
AGAP009973-RA	7
AGAP010133-RA	7
AGAP010229-RA	7
AGAP010249-RA	7
AGAP010258-RA	7
AGAP010368-RA	7
AGAP010386-RA	7
AGAP010481-RA	7
AGAP010543-RA	7
AGAP010568-RA	7
AGAP010638-RA	7
AGAP010714-RA	7
AGAP010719-RA	7
AGAP010843-RA	7
AGAP010875-RA	7
AGAP010983-RA	7
AGAP011044-RA	7
AGAP011051-RA	7
AGAP011104-RA	7
AGAP011107-RA	7
AGAP011190-RA	7
AGAP011286-RA	7
AGAP011447-RA	7
AGAP011535-RA	7
AGAP011551-RA	7
AGAP011580-RA	7
AGAP011754-RA	7
AGAP011766-RA	7
AGAP011798-RA	7
AGAP011800-RA	7
AGAP011940-RA	7
AGAP012148-RA	7
AGAP012168-RA	7
AGAP012185-RA	7
AGAP012281-RA	7
AGAP012292-RA	7
AGAP012307-RA	7
AGAP012398-RA	7
AGAP013310-RA	7
AGAP028617-RA	7
AGAP000023-RA	7
AGAP000092-RA	7
AGAP000304-RC	7
AGAP000375-RA	7
AGAP000378-RA	7
AGAP000824-RA	7
AGAP000958-RA	7

AGAP001032-RB	7
AGAP001107-RB	7
AGAP001136-RA	7
AGAP001200-RA	7
AGAP001209-RA	7
AGAP001639-RB	7
AGAP001651-RA	7
AGAP001721-RA	7
AGAP001791-RA	7
AGAP001870-RA	7
AGAP001899-RA	7
AGAP001958-RA	7
AGAP002054-RA	7
AGAP002113-RA	7
AGAP002166-RA	7
AGAP002169-RA	7
AGAP002414-RA	7
AGAP002428-RB	7
AGAP002509-RA	7
AGAP002564-RA	7
AGAP002586-RA	7
AGAP002606-RA	7
AGAP002783-RA	7
AGAP002788-RA	7
AGAP003023-RA	7
AGAP003043-RB	7
AGAP003167-RA	7
AGAP003192-RA	7
AGAP003200-RA	7
AGAP003358-RA	7
AGAP003397-RA	7
AGAP003492-RD	7
AGAP003615-RA	7
AGAP003794-RB	7
AGAP003857-RA	7
AGAP004096-RB	7
AGAP004109-RA	7
AGAP004332-RA	7
AGAP004372-RA	7
AGAP004394-RA	7
AGAP004451-RA	7
AGAP004502-RA	7
AGAP004598-RA	7
AGAP004798-RA	7
AGAP004867-RA	7
AGAP004868-RA	7
AGAP004990-RA	7
AGAP005082-RA	7
AGAP005108-RA	7
AGAP005121-RB	7
AGAP005401-RA	7
AGAP005425-RA	7
AGAP005531-RA	7
AGAP005645-RA	7

AGAP005749-RA	7
AGAP005782-RA	7
AGAP005894-RA	7
AGAP005929-RA	7
AGAP005971-RA	7
AGAP006040-RA	7
AGAP006096-RA	7
AGAP006228-RA	7
AGAP006243-RA	7
AGAP006522-RA	7
AGAP006686-RA	7
AGAP006755-RA	7
AGAP006766-RA	7
AGAP006996-RA	7
AGAP007088-RA	7
AGAP007106-RA	7
AGAP007113-RA	7
AGAP007131-RA	7
AGAP007567-RA	7
AGAP007584-RA	7
AGAP007620-RA	7
AGAP007843-RA	7
AGAP007902-RA	7
AGAP007941-RA	7
AGAP007970-RA	7
AGAP008007-RA	7
AGAP008219-RA	7
AGAP008252-RA	7
AGAP008444-RA	7
AGAP008606-RA	7
AGAP009051-RA	7
AGAP009119-RA	7
AGAP009146-RA	7
AGAP009271-RA	7
AGAP009388-RB	7
AGAP009945-RA	7
AGAP010073-RA	7
AGAP010074-RA	7
AGAP010193-RA	7
AGAP010428-RA	7
AGAP010445-RA	7
AGAP010517-RB	7
AGAP010718-RA	7
AGAP010769-RA	7
AGAP010794-RA	7
AGAP010815-RA	7
AGAP011079-RA	7
AGAP011105-RA	7
AGAP011141-RA	7
AGAP011315-RB	7
AGAP011444-RA	7
AGAP011663-RA	7
AGAP011700-RA	7
AGAP011727-RA	7

AGAP011824-RA	7
AGAP011827-RA	7
AGAP011849-RA	7
AGAP012038-RA	7
AGAP012339-RA	7
AGAP012577-RA	7
AGAP012705-RA	7
AGAP000022-RA	7
AGAP000278-RA	7
AGAP000625-RA	7
AGAP000626-RA	7
AGAP000818-RA	7
AGAP000862-RA	7
AGAP000883-RA	7
AGAP001229-RA	7
AGAP001273-RA	7
AGAP001377-RA	7
AGAP001645-RA	7
AGAP001759-RA	7
AGAP001973-RA	7
AGAP002278-RA	7
AGAP002356-RA	7
AGAP002396-RA	7
AGAP002652-RB	7
AGAP003008-RB	7
AGAP003016-RA	7
AGAP003088-RA	7
AGAP003243-RA	7
AGAP003367-RA	7
AGAP003405-RA	7
AGAP003457-RA	7
AGAP003533-RA	7
AGAP003687-RA	7
AGAP004197-RA	7
AGAP004458-RC	7
AGAP004629-RA	7
AGAP004786-RA	7
AGAP004960-RA	7
AGAP005246-RD	7
AGAP005535-RA	7
AGAP005617-RB	7
AGAP005618-RA	7
AGAP005672-RA	7
AGAP006607-RA	7
AGAP006733-RA	7
AGAP006962-RA	7
AGAP007474-RA	7
AGAP007538-RA	7
AGAP007619-RA	7
AGAP007786-RA	7
AGAP007938-RA	7
AGAP008501-RA	7
AGAP008708-RA	7
AGAP008762-RA	7

AGAP008837-RA	7
AGAP008908-RA	7
AGAP009057-RA	7
AGAP009265-RA	7
AGAP009647-RA	7
AGAP009653-RA	7
AGAP009865-RA	7
AGAP009893-RA	7
AGAP009897-RA	7
AGAP009919-RA	7
AGAP010010-RA	7
AGAP010321-RA	7
AGAP010421-RA	7
AGAP010727-RA	7
AGAP011130-RA	7
AGAP011157-RA	7
AGAP011564-RA	7
AGAP011805-RA	7
AGAP012014-RB	7
AGAP012191-RA	7
AGAP012302-RA	7
AGAP000618-RA	7
AGAP001256-RC	7
AGAP001760-RA	7
AGAP001802-RB	7
AGAP002047-RA	7
AGAP002440-RC	7
AGAP002969-RA	7
AGAP003069-RA	7
AGAP003168-RA	7
AGAP003711-RA	7
AGAP004055-RB	7
AGAP004142-RA	7
AGAP004159-RB	7
AGAP004263-RA	7
AGAP004484-RA	7
AGAP004528-RA	7
AGAP004787-RA	7
AGAP004852-RA	7
AGAP005234-RA	7
AGAP005316-RA	7
AGAP005520-RA	7
AGAP005529-RB	7
AGAP005961-RA	7
AGAP006456-RA	7
AGAP006613-RB	7
AGAP007161-RA	7
AGAP007309-RA	7
AGAP008268-RA	7
AGAP008288-RA	7
AGAP008667-RA	7
AGAP009212-RA	7
AGAP009246-RA	7
AGAP009602-RA	7

AGAP010968-RA	7
AGAP011050-RA	7
AGAP011166-RA	7
AGAP028621-RA	7
AGAP000165-RA	7
AGAP000897-RA	7
AGAP001325-RA	7
AGAP001341-RA	7
AGAP001459-RC	7
AGAP001884-RA	7
AGAP003319-RA	7
AGAP003584-RA	7
AGAP003722-RA	7
AGAP004396-RA	7
AGAP004750-RA	7
AGAP005109-RA	7
AGAP005662-RA	7
AGAP006615-RA	7
AGAP006918-RA	7
AGAP007525-RA	7
AGAP007684-RB	7
AGAP007711-RA	7
AGAP008816-RA	7
AGAP008931-RA	7
AGAP009105-RA	7
AGAP009110-RA	7
AGAP009443-RA	7
AGAP009685-RA	7
AGAP010214-RA	7
AGAP010572-RA	7
AGAP011053-RA	7
AGAP012189-RA	7
AGAP012352-RA	7
AGAP028491-RA	7
AGAP000654-RA	7
AGAP001995-RA	7
AGAP002374-RA	7
AGAP002499-RA	7
AGAP002569-RA	7
AGAP002769-RA	7
AGAP002879-RB	7
AGAP003021-RB	7
AGAP003176-RA	7
AGAP004164-RA	7
AGAP007082-RA	7
AGAP007633-RD	7
AGAP007867-RA	7
AGAP008020-RA	7
AGAP008763-RB	7
AGAP008884-RA	7
AGAP009072-RA	7
AGAP009788-RA	7
AGAP010464-RA	7
AGAP010964-RA	7

AGAP011438-RA	7
AGAP011464-RA	7
AGAP012140-RA	7
AGAP012167-RA	7
AGAP000167-RA	7
AGAP000877-RA	7
AGAP002340-RA	7
AGAP002655-RA	7
AGAP002931-RB	7
AGAP004486-RA	7
AGAP004616-RA	7
AGAP005327-RA	7
AGAP005948-RA	7
AGAP006821-RA	7
AGAP007055-RA	7
AGAP007593-RA	7
AGAP008731-RA	7
AGAP008802-RA	7
AGAP008985-RA	7
AGAP010310-RA	7
AGAP011172-RA	7
AGAP011478-RA	7
AGAP012048-RA	7
AGAP012053-RA	7
AGAP001587-RA	7
AGAP001713-RD	7
AGAP003250-RA	7
AGAP004146-RA	7
AGAP004551-RA	7
AGAP005655-RA	7
AGAP005775-RA	7
AGAP007975-RB	7
AGAP008046-RA	7
AGAP009883-RA	7
AGAP011426-RA	7
AGAP012251-RA	7
AGAP000721-RA	7
AGAP001420-RA	7
AGAP002889-RA	7
AGAP008645-RA	7
AGAP009604-RA	7
AGAP010364-RA	7
AGAP010429-RA	7
AGAP011159-RA	7
AGAP002328-RA	7
AGAP005630-RA	7
AGAP006360-RA	7
AGAP028568-RA	7
AGAP003769-RA	7
AGAP009333-RA	7
AGAP001312-RA	7
AGAP005621-RA	7
AGAP010156-RA	7
AGAP000655-RA	7

AGAP002464-RA	7
AGAP004199-RA	7
AGAP002093-RA	7
AGAP002170-RA	7
AGAP003553-RA	7
AGAP003725-RB	7
AGAP007642-RA	7
AGAP007921-RA	7
AGAP010150-RA	7
AGAP003119-RA	7
AGAP000749-RA	7
AGAP002245-RA	7
AGAP004164-RB	7
AGAP004366-RA	7
AGAP007574-RA	7
AGAP008387-RA	7
AGAP008653-RA	7
AGAP010557-RA	7
AGAP000669-RA	7
AGAP002182-RA	7
AGAP011687-RA	7
AGAP013412-RA	8
AGAP000633-RA	8
AGAP000888-RB	8
AGAP001307-RA	8
AGAP002341-RA	8
AGAP002387-RA	8
AGAP002571-RA	8
AGAP002657-RA	8
AGAP002661-RA	8
AGAP002812-RA	8
AGAP002956-RA	8
AGAP003212-RA	8
AGAP003229-RA	8
AGAP003324-RA	8
AGAP003425-RA	8
AGAP003475-RB	8
AGAP003744-RA	8
AGAP003968-RA	8
AGAP004056-RA	8
AGAP004063-RA	8
AGAP004094-RA	8
AGAP004110-RA	8
AGAP004513-RB	8
AGAP004585-RA	8
AGAP004910-RA	8
AGAP005118-RA	8
AGAP005148-RA	8
AGAP005600-RA	8
AGAP005824-RA	8
AGAP006051-RA	8
AGAP006163-RA	8
AGAP006706-RA	8
AGAP006952-RA	8

AGAP007297-RA	8
AGAP007383-RA	8
AGAP007477-RA	8
AGAP008196-RA	8
AGAP008298-RA	8
AGAP008424-RA	8
AGAP008770-RA	8
AGAP009012-RA	8
AGAP009019-RA	8
AGAP009163-RA	8
AGAP009613-RA	8
AGAP009614-RA	8
AGAP009800-RA	8
AGAP010212-RA	8
AGAP010443-RA	8
AGAP011055-RA	8
AGAP011081-RA	8
AGAP011402-RA	8
AGAP011636-RA	8
AGAP011661-RA	8
AGAP012030-RC	8
AGAP012283-RA	8
AGAP013136-RA	8
AGAP013212-RA	8
AGAP028146-RD	8
AGAP028510-RA	8
AGAP000044-RA	8
AGAP000666-RA	8
AGAP000813-RA	8
AGAP001092-RA	8
AGAP001612-RA	8
AGAP001735-RA	8
AGAP001896-RA	8
AGAP002088-RA	8
AGAP002467-RA	8
AGAP002607-RA	8
AGAP002759-RA	8
AGAP002880-RA	8
AGAP003112-RA	8
AGAP003580-RA	8
AGAP003810-RA	8
AGAP004382-RA	8
AGAP004904-RA	8
AGAP005861-RB	8
AGAP005947-RA	8
AGAP006278-RA	8
AGAP006603-RA	8
AGAP007864-RC	8
AGAP008249-RA	8
AGAP008345-RA	8
AGAP009512-RA	8
AGAP009682-RA	8
AGAP011114-RA	8
AGAP013296-RA	8

AGAP003360-RA	8
AGAP003935-RA	8
AGAP007384-RA	8
AGAP009182-RA	8
AGAP000042-RA	8
AGAP000425-RA	8
AGAP002659-RA	8
AGAP011757-RA	8
AGAP012246-RA	8
AGAP008475-RA	8
AGAP007245-RA	8
AGAP002713-RA	8
AGAP000896-RA	8
AGAP002906-RA	8
AGAP011534-RA	8
AGAP010139-RD	9
AGAP011833-RA	9
AGAP002996-RD	9
AGAP003164-RA	9
AGAP003592-RB	9
AGAP004238-RA	9
AGAP005891-RB	9
AGAP005910-RB	9
AGAP005972-RB	9
AGAP005984-RB	9
AGAP007784-RB	9
AGAP009658-RA	9
AGAP009842-RA	9
AGAP010130-RB	9
AGAP010691-RA	9
AGAP010929-RA	9
AGAP011521-RA	9
AGAP011531-RA	9
AGAP012135-RA	9
AGAP012407-RA	9
AGAP012731-RA	9
AGAP013511-RC	9
AGAP028093-RA	9
AGAP000577-RA	9
AGAP001440-RB	9
AGAP001446-RD	9
AGAP001813-RB	9
AGAP003116-RA	9
AGAP003277-RD	9
AGAP003662-RB	9
AGAP003843-RA	9
AGAP004098-RB	9
AGAP004235-RC	9
AGAP004298-RC	9
AGAP004559-RK	9
AGAP005175-RB	9
AGAP005432-RA	9
AGAP005504-RA	9
AGAP005552-RB	9

AGAP005627-RB	9
AGAP005890-RA	9
AGAP006471-RA	9
AGAP007780-RB	9
AGAP007806-RC	9
AGAP007901-RG	9
AGAP008113-RB	9
AGAP008329-RC	9
AGAP008432-RD	9
AGAP008693-RA	9
AGAP009447-RB	9
AGAP010163-RA	9
AGAP010164-RC	9
AGAP010172-RB	9
AGAP010174-RA	9
AGAP010792-RA	9
AGAP012045-RA	9
AGAP000095-RA	9
AGAP000969-RD	9
AGAP001174-RA	9
AGAP001267-RA	9
AGAP001653-RB	9
AGAP002076-RA	9
AGAP002925-RC	9
AGAP003768-RB	9
AGAP004362-RE	9
AGAP004819-RB	9
AGAP004846-RC	9
AGAP005091-RE	9
AGAP006036-RA	9
AGAP007366-RB	9
AGAP011424-RA	9
AGAP013731-RA	9
AGAP009431-RB	9
AGAP008276-RA	9
AGAP004212-RA	9
AGAP006353-RA	9
AGAP006921-RA	9
AGAP000986-RA	10
AGAP007069-RA	10
AGAP008165-RF	10
AGAP008311-RA	10
AGAP010230-RA	10
AGAP000188-RB	10
AGAP000466-RA	10
AGAP000885-RA	10
AGAP001010-RA	10
AGAP001983-RA	10
AGAP002157-RA	10
AGAP003026-RA	10
AGAP003191-RA	10
AGAP003606-RA	10
AGAP003934-RA	10
AGAP003937-RA	10

AGAP004450-RB	10
AGAP004834-RB	10
AGAP006369-RA	10
AGAP006582-RC	10
AGAP006906-RA	10
AGAP007061-RA	10
AGAP007249-RA	10
AGAP008203-RA	10
AGAP009283-RA	10
AGAP009935-RA	10
AGAP010311-RA	10
AGAP011516-RA	10
AGAP011642-RA	10
AGAP011708-RA	10
AGAP011948-RA	10
AGAP012828-RA	10
AGAP028025-RA	10
AGAP000798-RA	10
AGAP003067-RA	10
AGAP006375-RA	10
AGAP006490-RA	10
AGAP006835-RB	10
AGAP008144-RB	10
AGAP008892-RA	10
AGAP009768-RA	10
AGAP010436-RA	10
AGAP011749-RA	10
AGAP028061-RA	10
AGAP000163-RB	10
AGAP000393-RB	10
AGAP000882-RA	10
AGAP001213-RA	10
AGAP001336-RA	10
AGAP002270-RA	10
AGAP002638-RA	10
AGAP002835-RA	10
AGAP002989-RA	10
AGAP002999-RA	10
AGAP003712-RA	10
AGAP004017-RA	10
AGAP004237-RA	10
AGAP004761-RA	10
AGAP005235-RA	10
AGAP006441-RA	10
AGAP006593-RA	10
AGAP006665-RC	10
AGAP007086-RA	10
AGAP007760-RA	10
AGAP008435-RA	10
AGAP008636-RA	10
AGAP008730-RA	10
AGAP008878-RA	10
AGAP009098-RA	10
AGAP009239-RA	10

AGAP009284-RA	10
AGAP009703-RA	10
AGAP009978-RA	10
AGAP010157-RB	10
AGAP010238-RA	10
AGAP010280-RA	10
AGAP010633-RA	10
AGAP010701-RA	10
AGAP010806-RA	10
AGAP010928-RA	10
AGAP011296-RA	10
AGAP011498-RA	10
AGAP011505-RA	10
AGAP011515-RA	10
AGAP011936-RA	10
AGAP012964-RA	10
AGAP013509-RA	10
AGAP000107-RA	10
AGAP000129-RA	10
AGAP000245-RA	10
AGAP000359-RA	10
AGAP000520-RA	10
AGAP000521-RA	10
AGAP000540-RA	10
AGAP000553-RA	10
AGAP000676-RA	10
AGAP001236-RA	10
AGAP001506-RA	10
AGAP001960-RA	10
AGAP002291-RA	10
AGAP002691-RA	10
AGAP002726-RA	10
AGAP003751-RA	10
AGAP005837-RA	10
AGAP005992-RA	10
AGAP006023-RA	10
AGAP006257-RA	10
AGAP006382-RA	10
AGAP007382-RA	10
AGAP009100-RA	10
AGAP009191-RA	10
AGAP009695-RA	10
AGAP010878-RA	10
AGAP012447-RA	10
AGAP028039-RA	10
AGAP002238-RB	10
AGAP002262-RA	10
AGAP004161-RA	10
AGAP004455-RA	10
AGAP005815-RA	10
AGAP006757-RA	10
AGAP008575-RA	10
AGAP008584-RA	10
AGAP009622-RA	10

AGAP028212-RA	10
AGAP009353-RA	10
AGAP000748-RB	10
AGAP001113-RA	10
AGAP001557-RA	10
AGAP002695-RA	10
AGAP011357-RB	10
AGAP004858-RA	10
AGAP012171-RA	10
AGAP012364-RA	10
AGAP006258-RA	10
AGAP012320-RA	10
AGAP000182-RA	10
AGAP000162-RA	10
AGAP012616-RA	10
AGAP010385-RA	10
AGAP009533-RA	10
AGAP003374-RA	10
AGAP011812-RA	10
AGAP006111-RA	10
AGAP003873-RA	10
AGAP004784-RA	10
AGAP011342-RA	10
AGAP000560-RA	11
AGAP001858-RA	11
AGAP003925-RA	11
AGAP005006-RA	11
AGAP006730-RA	11
AGAP006965-RA	11
AGAP007576-RA	11
AGAP007586-RA	11
AGAP007618-RA	11
AGAP007737-RA	11
AGAP007831-RA	11
AGAP008332-RA	11
AGAP008631-RB	11
AGAP008797-RA	11
AGAP008925-RA	11
AGAP009067-RA	11
AGAP009167-RA	11
AGAP009296-RA	11
AGAP009434-RA	11
AGAP009436-RA	11
AGAP009643-RA	11
AGAP009724-RA	11
AGAP010211-RA	11
AGAP010217-RA	11
AGAP010218-RA	11
AGAP010234-RA	11
AGAP010322-RA	11
AGAP010379-RA	11
AGAP010566-RA	11
AGAP010643-RA	11
AGAP010644-RA	11

AGAP010723-RA	11
AGAP010738-RA	11
AGAP010768-RA	11
AGAP011152-RA	11
AGAP011350-RA	11
AGAP011463-RA	11
AGAP011503-RA	11
AGAP011822-RA	11
AGAP011893-RA	11
AGAP011904-RA	11
AGAP012046-RA	11
AGAP012229-RA	11
AGAP012416-RA	11
AGAP013023-RA	11
AGAP028511-RA	11
AGAP028628-RA	11
AGAP028632-RA	11
AGAP028649-RA	11
AGAP000011-RA	11
AGAP000149-RA	11
AGAP000161-RA	11
AGAP000195-RA	11
AGAP000211-RA	11
AGAP000307-RA	11
AGAP000711-RA	11
AGAP000719-RA	11
AGAP000728-RB	11
AGAP000765-RA	11
AGAP000791-RA	11
AGAP000842-RA	11
AGAP000995-RA	11
AGAP001141-RB	11
AGAP001228-RB	11
AGAP001268-RA	11
AGAP001271-RB	11
AGAP001363-RA	11
AGAP001391-RA	11
AGAP001423-RA	11
AGAP001470-RA	11
AGAP001699-RA	11
AGAP001704-RA	11
AGAP001744-RA	11
AGAP001887-RA	11
AGAP001953-RA	11
AGAP002118-RA	11
AGAP002128-RB	11
AGAP002227-RA	11
AGAP002286-RA	11
AGAP002357-RA	11
AGAP002477-RA	11
AGAP002498-RA	11
AGAP002501-RA	11
AGAP002530-RA	11
AGAP002538-RA	11

AGAP002644-RA	11
AGAP002729-RA	11
AGAP002816-RA	11
AGAP002948-RA	11
AGAP002961-RA	11
AGAP002968-RA	11
AGAP003162-RA	11
AGAP003207-RA	11
AGAP003265-RA	11
AGAP003311-RA	11
AGAP003373-RA	11
AGAP003437-RA	11
AGAP003537-RB	11
AGAP003614-RA	11
AGAP003629-RA	11
AGAP003636-RA	11
AGAP003643-RA	11
AGAP003715-RA	11
AGAP003716-RA	11
AGAP003719-RA	11
AGAP003728-RA	11
AGAP003808-RA	11
AGAP003818-RB	11
AGAP003844-RA	11
AGAP003856-RA	11
AGAP003867-RA	11
AGAP004089-RB	11
AGAP004136-RA	11
AGAP004143-RA	11
AGAP004176-RA	11
AGAP004204-RA	11
AGAP004207-RA	11
AGAP004226-RA	11
AGAP004255-RA	11
AGAP004271-RA	11
AGAP004371-RA	11
AGAP004374-RA	11
AGAP004417-RA	11
AGAP004434-RA	11
AGAP004448-RB	11
AGAP004466-RA	11
AGAP004469-RA	11
AGAP004477-RA	11
AGAP004510-RA	11
AGAP004560-RA	11
AGAP004749-RB	11
AGAP004754-RA	11
AGAP004780-RA	11
AGAP004845-RA	11
AGAP004932-RA	11
AGAP005040-RA	11
AGAP005044-RA	11
AGAP005210-RA	11
AGAP005282-RA	11

AGAP005299-RA	11
AGAP005382-RA	11
AGAP005402-RA	11
AGAP005419-RA	11
AGAP005499-RA	11
AGAP005506-RA	11
AGAP005561-RA	11
AGAP005701-RA	11
AGAP005719-RA	11
AGAP005748-RA	11
AGAP005825-RA	11
AGAP005860-RA	11
AGAP005883-RA	11
AGAP005892-RA	11
AGAP005893-RA	11
AGAP005931-RA	11
AGAP005940-RA	11
AGAP005979-RA	11
AGAP006031-RA	11
AGAP006193-RA	11
AGAP006205-RA	11
AGAP006232-RA	11
AGAP006269-RA	11
AGAP006280-RA	11
AGAP006427-RA	11
AGAP006517-RA	11
AGAP006574-RA	11
AGAP006601-RB	11
AGAP006682-RA	11
AGAP006784-RA	11
AGAP006808-RA	11
AGAP006884-RA	11
AGAP006888-RA	11
AGAP006933-RA	11
AGAP006969-RA	11
AGAP006998-RA	11
AGAP007010-RA	11
AGAP007022-RA	11
AGAP007027-RA	11
AGAP007085-RA	11
AGAP007097-RA	11
AGAP007332-RA	11
AGAP007504-RA	11
AGAP007522-RA	11
AGAP007535-RA	11
AGAP007608-RA	11
AGAP007638-RA	11
AGAP007778-RA	11
AGAP007863-RA	11
AGAP007891-RA	11
AGAP007967-RA	11
AGAP007969-RA	11
AGAP007998-RA	11
AGAP008085-RA	11

AGAP008094-RA	11
AGAP008107-RA	11
AGAP008236-RA	11
AGAP008344-RA	11
AGAP008350-RA	11
AGAP008379-RA	11
AGAP008389-RA	11
AGAP008484-RA	11
AGAP008585-RA	11
AGAP008626-RA	11
AGAP008639-RA	11
AGAP008652-RA	11
AGAP008721-RA	11
AGAP008736-RA	11
AGAP008737-RA	11
AGAP008805-RA	11
AGAP008806-RA	11
AGAP008885-RA	11
AGAP009023-RA	11
AGAP009034-RA	11
AGAP009071-RA	11
AGAP009073-RA	11
AGAP009154-RA	11
AGAP009432-RA	11
AGAP009445-RA	11
AGAP009558-RA	11
AGAP009776-RA	11
AGAP009913-RA	11
AGAP009972-RA	11
AGAP010012-RA	11
AGAP010137-RA	11
AGAP010197-RB	11
AGAP010228-RA	11
AGAP010396-RA	11
AGAP010510-RA	11
AGAP010514-RA	11
AGAP010534-RA	11
AGAP010626-RA	11
AGAP010677-RA	11
AGAP010877-RA	11
AGAP010917-RA	11
AGAP011057-RA	11
AGAP011108-RA	11
AGAP011147-RA	11
AGAP011158-RA	11
AGAP011199-RA	11
AGAP011204-RA	11
AGAP011244-RA	11
AGAP011456-RA	11
AGAP011471-RA	11
AGAP011474-RA	11
AGAP011570-RA	11
AGAP011587-RA	11
AGAP011730-RA	11

AGAP011975-RA	11
AGAP012084-RA	11
AGAP012089-RA	11
AGAP012096-RA	11
AGAP012113-RA	11
AGAP012115-RA	11
AGAP012139-RA	11
AGAP012247-RA	11
AGAP012248-RA	11
AGAP012386-RA	11
AGAP012404-RA	11
AGAP013516-RB	11
AGAP028394-RA	11
AGAP000046-RA	11
AGAP000102-RA	11
AGAP000173-RA	11
AGAP000187-RA	11
AGAP000346-RA	11
AGAP000388-RA	11
AGAP000406-RA	11
AGAP000412-RA	11
AGAP000434-RA	11
AGAP000456-RA	11
AGAP000615-RA	11
AGAP000660-RA	11
AGAP000727-RA	11
AGAP000947-RA	11
AGAP001027-RA	11
AGAP001037-RA	11
AGAP001058-RA	11
AGAP001068-RA	11
AGAP001110-RB	11
AGAP001144-RA	11
AGAP001357-RA	11
AGAP001364-RA	11
AGAP001418-RA	11
AGAP001421-RA	11
AGAP001467-RA	11
AGAP001469-RA	11
AGAP001519-RA	11
AGAP001527-RA	11
AGAP001724-RA	11
AGAP001734-RA	11
AGAP001738-RA	11
AGAP001820-RA	11
AGAP001891-RA	11
AGAP001894-RA	11
AGAP001941-RA	11
AGAP001947-RA	11
AGAP002081-RA	11
AGAP002086-RA	11
AGAP002123-RA	11
AGAP002147-RA	11
AGAP002194-RA	11

AGAP002251-RA	11
AGAP002300-RB	11
AGAP002371-RA	11
AGAP002557-RA	11
AGAP002594-RA	11
AGAP002662-RA	11
AGAP002721-RA	11
AGAP002736-RA	11
AGAP002800-RA	11
AGAP002939-RB	11
AGAP002982-RA	11
AGAP003003-RA	11
AGAP003006-RA	11
AGAP003014-RA	11
AGAP003126-RA	11
AGAP003346-RA	11
AGAP003366-RA	11
AGAP003389-RA	11
AGAP003408-RA	11
AGAP003417-RB	11
AGAP003422-RA	11
AGAP003429-RB	11
AGAP003489-RA	11
AGAP003551-RA	11
AGAP003571-RC	11
AGAP003638-RA	11
AGAP003650-RA	11
AGAP003661-RB	11
AGAP003779-RA	11
AGAP004026-RB	11
AGAP004049-RA	11
AGAP004144-RA	11
AGAP004268-RA	11
AGAP004402-RA	11
AGAP004420-RA	11
AGAP004439-RA	11
AGAP004699-RA	11
AGAP004703-RA	11
AGAP004746-RA	11
AGAP004801-RC	11
AGAP004854-RA	11
AGAP004864-RA	11
AGAP004881-RA	11
AGAP005003-RA	11
AGAP005053-RA	11
AGAP005188-RA	11
AGAP005405-RA	11
AGAP005567-RA	11
AGAP005700-RA	11
AGAP005736-RA	11
AGAP005759-RA	11
AGAP005863-RA	11
AGAP005869-RA	11
AGAP005945-RA	11

AGAP005954-RA	11
AGAP005956-RA	11
AGAP006226-RA	11
AGAP006249-RA	11
AGAP006413-RA	11
AGAP006819-RA	11
AGAP006890-RB	11
AGAP006947-RA	11
AGAP006992-RA	11
AGAP006993-RA	11
AGAP007057-RA	11
AGAP007475-RA	11
AGAP007499-RA	11
AGAP007579-RA	11
AGAP007765-RA	11
AGAP007796-RA	11
AGAP007828-RA	11
AGAP007865-RA	11
AGAP007870-RA	11
AGAP008026-RA	11
AGAP008089-RA	11
AGAP008150-RA	11
AGAP008156-RA	11
AGAP008181-RA	11
AGAP008212-RA	11
AGAP008315-RA	11
AGAP008392-RA	11
AGAP008406-RA	11
AGAP008601-RA	11
AGAP008620-RA	11
AGAP008641-RA	11
AGAP008642-RA	11
AGAP008662-RA	11
AGAP008669-RA	11
AGAP008879-RA	11
AGAP008990-RA	11
AGAP009029-RA	11
AGAP009037-RA	11
AGAP009059-RA	11
AGAP009143-RA	11
AGAP009255-RA	11
AGAP009405-RA	11
AGAP009490-RA	11
AGAP009509-RA	11
AGAP009544-RA	11
AGAP009571-RA	11
AGAP009603-RA	11
AGAP009765-RA	11
AGAP009831-RA	11
AGAP009841-RA	11
AGAP009896-RA	11
AGAP009921-RA	11
AGAP009942-RA	11
AGAP009954-RA	11

AGAP010273-RA	11
AGAP010339-RA	11
AGAP010403-RA	11
AGAP010681-RA	11
AGAP010725-RA	11
AGAP010935-RA	11
AGAP011036-RA	11
AGAP011140-RA	11
AGAP011149-RA	11
AGAP011189-RA	11
AGAP011245-RA	11
AGAP011247-RA	11
AGAP011292-RA	11
AGAP011685-RA	11
AGAP011771-RA	11
AGAP011801-RA	11
AGAP011841-RA	11
AGAP011859-RA	11
AGAP012005-RA	11
AGAP012499-RA	11
AGAP012792-RA	11
AGAP013013-RA	11
AGAP013266-RA	11
AGAP013357-RA	11
AGAP013519-RA	11
AGAP028011-RA	11
AGAP028559-RA	11
AGAP000002-RA	11
AGAP000007-RA	11
AGAP000090-RA	11
AGAP000322-RA	11
AGAP000396-RA	11
AGAP000439-RA	11
AGAP000700-RC	11
AGAP000770-RA	11
AGAP000880-RC	11
AGAP000886-RA	11
AGAP001102-RA	11
AGAP001140-RA	11
AGAP001253-RA	11
AGAP001376-RA	11
AGAP001473-RA	11
AGAP001500-RA	11
AGAP001549-RA	11
AGAP001719-RA	11
AGAP001746-RA	11
AGAP001827-RA	11
AGAP002039-RA	11
AGAP002092-RA	11
AGAP002101-RA	11
AGAP002198-RA	11
AGAP002268-RA	11
AGAP002277-RA	11
AGAP002491-RA	11

AGAP002626-RA	11
AGAP002653-RA	11
AGAP002781-RA	11
AGAP002923-RA	11
AGAP003040-RA	11
AGAP003185-RA	11
AGAP003199-RA	11
AGAP003202-RA	11
AGAP003350-RB	11
AGAP003371-RA	11
AGAP003395-RA	11
AGAP003548-RA	11
AGAP003557-RA	11
AGAP003596-RA	11
AGAP003730-RA	11
AGAP004102-RA	11
AGAP004145-RA	11
AGAP004234-RA	11
AGAP004317-RA	11
AGAP004404-RB	11
AGAP004546-RA	11
AGAP004606-RA	11
AGAP004653-RA	11
AGAP004709-RA	11
AGAP004745-RA	11
AGAP004747-RA	11
AGAP004861-RA	11
AGAP004992-RA	11
AGAP005062-RA	11
AGAP005261-RA	11
AGAP005287-RA	11
AGAP005430-RA	11
AGAP005523-RA	11
AGAP005545-RA	11
AGAP005560-RA	11
AGAP005581-RA	11
AGAP005712-RA	11
AGAP005953-RA	11
AGAP006044-RA	11
AGAP006100-RA	11
AGAP006116-RA	11
AGAP006354-RA	11
AGAP006445-RA	11
AGAP006895-RA	11
AGAP006943-RA	11
AGAP007143-RA	11
AGAP007181-RA	11
AGAP007308-RA	11
AGAP007592-RA	11
AGAP007600-RA	11
AGAP007774-RA	11
AGAP007911-RA	11
AGAP008074-RA	11
AGAP008251-RA	11

AGAP008312-RA	11
AGAP008497-RA	11
AGAP008634-RA	11
AGAP008664-RA	11
AGAP008906-RA	11
AGAP008915-RA	11
AGAP008932-RA	11
AGAP009180-RA	11
AGAP009498-RA	11
AGAP009839-RA	11
AGAP009840-RA	11
AGAP010041-RA	11
AGAP010066-RA	11
AGAP010070-RA	11
AGAP010275-RA	11
AGAP010459-RA	11
AGAP010494-RA	11
AGAP010687-RA	11
AGAP010700-RA	11
AGAP010937-RA	11
AGAP011133-RA	11
AGAP011583-RA	11
AGAP011654-RA	11
AGAP011689-RA	11
AGAP011705-RA	11
AGAP011723-RA	11
AGAP011765-RA	11
AGAP011820-RA	11
AGAP012134-RA	11
AGAP012136-RA	11
AGAP012282-RA	11
AGAP012513-RA	11
AGAP013747-RB	11
AGAP028123-RA	11
AGAP028620-RA	11
AGAP000035-RA	11
AGAP000118-RA	11
AGAP000142-RA	11
AGAP000175-RA	11
AGAP000180-RA	11
AGAP000285-RA	11
AGAP000376-RA	11
AGAP000403-RA	11
AGAP000653-RA	11
AGAP000698-RA	11
AGAP000717-RA	11
AGAP000761-RA	11
AGAP001320-RA	11
AGAP001475-RA	11
AGAP001621-RB	11
AGAP001743-RA	11
AGAP002036-RA	11
AGAP002472-RA	11
AGAP002549-RA	11

AGAP002645-RA	11
AGAP002706-RA	11
AGAP002752-RA	11
AGAP002779-RA	11
AGAP002908-RA	11
AGAP002920-RA	11
AGAP003018-RA	11
AGAP003039-RA	11
AGAP003048-RA	11
AGAP003264-RA	11
AGAP003317-RA	11
AGAP003343-RA	11
AGAP003478-RA	11
AGAP003521-RA	11
AGAP003576-RA	11
AGAP003605-RA	11
AGAP003613-RA	11
AGAP003644-RA	11
AGAP003834-RA	11
AGAP004194-RB	11
AGAP004301-RA	11
AGAP004520-RA	11
AGAP004725-RA	11
AGAP004737-RA	11
AGAP004873-RA	11
AGAP004953-RA	11
AGAP005080-RA	11
AGAP005236-RA	11
AGAP005350-RA	11
AGAP005376-RA	11
AGAP005521-RA	11
AGAP005551-RA	11
AGAP005632-RA	11
AGAP005826-RA	11
AGAP005856-RA	11
AGAP005897-RA	11
AGAP005989-RA	11
AGAP006357-RA	11
AGAP006472-RA	11
AGAP006548-RA	11
AGAP006570-RA	11
AGAP006650-RA	11
AGAP006678-RA	11
AGAP006750-RA	11
AGAP006777-RA	11
AGAP006878-RA	11
AGAP006939-RA	11
AGAP006949-RA	11
AGAP007000-RA	11
AGAP007217-RA	11
AGAP007545-RA	11
AGAP007565-RB	11
AGAP007629-RB	11
AGAP007644-RA	11

AGAP007683-RA	11
AGAP007722-RA	11
AGAP007920-RA	11
AGAP008053-RA	11
AGAP008157-RA	11
AGAP008184-RA	11
AGAP008518-RA	11
AGAP008768-RA	11
AGAP008804-RB	11
AGAP009166-RA	11
AGAP009197-RA	11
AGAP009209-RA	11
AGAP009234-RA	11
AGAP009625-RA	11
AGAP010011-RA	11
AGAP010432-RA	11
AGAP010496-RB	11
AGAP010539-RA	11
AGAP010739-RA	11
AGAP011532-RA	11
AGAP011571-RA	11
AGAP011840-RA	11
AGAP012108-RA	11
AGAP012154-RA	11
AGAP028157-RA	11
AGAP028693-RA	11
AGAP000097-RA	11
AGAP000249-RA	11
AGAP001153-RA	11
AGAP001264-RA	11
AGAP001452-RA	11
AGAP001486-RA	11
AGAP001490-RA	11
AGAP001905-RB	11
AGAP002067-RA	11
AGAP002084-RA	11
AGAP002171-RA	11
AGAP002193-RA	11
AGAP002670-RA	11
AGAP002693-RA	11
AGAP002758-RA	11
AGAP002943-RA	11
AGAP003369-RA	11
AGAP003440-RA	11
AGAP003678-RA	11
AGAP003700-RA	11
AGAP003734-RA	11
AGAP003839-RA	11
AGAP003853-RA	11
AGAP004251-RB	11
AGAP004300-RA	11
AGAP004333-RA	11
AGAP004407-RA	11
AGAP004532-RA	11

AGAP004609-RA	11
AGAP004612-RA	11
AGAP005029-RB	11
AGAP005042-RA	11
AGAP005291-RA	11
AGAP005421-RA	11
AGAP005532-RA	11
AGAP005550-RA	11
AGAP005639-RA	11
AGAP006647-RA	11
AGAP006683-RC	11
AGAP006885-RA	11
AGAP007001-RA	11
AGAP007039-RA	11
AGAP007253-RA	11
AGAP007381-RA	11
AGAP007543-RA	11
AGAP007634-RA	11
AGAP007725-RA	11
AGAP007742-RA	11
AGAP008393-RA	11
AGAP008411-RA	11
AGAP008491-RA	11
AGAP008541-RA	11
AGAP008637-RA	11
AGAP009438-RA	11
AGAP009745-RA	11
AGAP009992-RA	11
AGAP010556-RA	11
AGAP011110-RA	11
AGAP011150-RA	11
AGAP011214-RA	11
AGAP011271-RA	11
AGAP011830-RA	11
AGAP013167-RA	11
AGAP000172-RA	11
AGAP000498-RB	11
AGAP000534-RB	11
AGAP000808-RA	11
AGAP000855-RA	11
AGAP000994-RA	11
AGAP001207-RA	11
AGAP001211-RA	11
AGAP001298-RA	11
AGAP001902-RA	11
AGAP002071-RA	11
AGAP002185-RA	11
AGAP002215-RA	11
AGAP002536-RA	11
AGAP002883-RA	11
AGAP003133-RA	11
AGAP003415-RA	11
AGAP003560-RA	11
AGAP003581-RA	11

AGAP003824-RA	11
AGAP004210-RA	11
AGAP004519-RA	11
AGAP004708-RA	11
AGAP005039-RA	11
AGAP005178-RA	11
AGAP005374-RA	11
AGAP005622-RA	11
AGAP006017-RA	11
AGAP006121-RA	11
AGAP006474-RA	11
AGAP006818-RA	11
AGAP007019-RA	11
AGAP007306-RA	11
AGAP007647-RA	11
AGAP007873-RA	11
AGAP008830-RA	11
AGAP009081-RA	11
AGAP009194-RA	11
AGAP009908-RA	11
AGAP010392-RA	11
AGAP011156-RA	11
AGAP011413-RA	11
AGAP011563-RA	11
AGAP011811-RA	11
AGAP012335-RA	11
AGAP013207-RA	11
AGAP013218-RA	11
AGAP013277-RA	11
AGAP028640-RA	11
AGAP000238-RA	11
AGAP000306-RA	11
AGAP000367-RA	11
AGAP000416-RB	11
AGAP000662-RC	11
AGAP000671-RA	11
AGAP001501-RA	11
AGAP001939-RA	11
AGAP002417-RA	11
AGAP002608-RA	11
AGAP002619-RA	11
AGAP002730-RA	11
AGAP003017-RA	11
AGAP003179-RA	11
AGAP003388-RA	11
AGAP003823-RB	11
AGAP005170-RA	11
AGAP005771-RA	11
AGAP006139-RB	11
AGAP006247-RA	11
AGAP007023-RA	11
AGAP007264-RA	11
AGAP007552-RA	11
AGAP007881-RA	11

AGAP009150-RA	11
AGAP009563-RA	11
AGAP009907-RA	11
AGAP010046-RA	11
AGAP010530-RA	11
AGAP010590-RA	11
AGAP010696-RA	11
AGAP010823-RA	11
AGAP012146-RA	11
AGAP012413-RA	11
AGAP012632-RA	11
AGAP028425-RA	11
AGAP028609-RA	11
AGAP000693-RA	11
AGAP000891-RA	11
AGAP001318-RA	11
AGAP002390-RA	11
AGAP003050-RA	11
AGAP003221-RA	11
AGAP003289-RA	11
AGAP003409-RA	11
AGAP003416-RA	11
AGAP003424-RA	11
AGAP004236-RA	11
AGAP004757-RA	11
AGAP004993-RA	11
AGAP005462-RA	11
AGAP006113-RA	11
AGAP006344-RA	11
AGAP006569-RA	11
AGAP006817-RA	11
AGAP007018-RA	11
AGAP007130-RA	11
AGAP007888-RA	11
AGAP007958-RA	11
AGAP008286-RA	11
AGAP008321-RA	11
AGAP008494-RB	11
AGAP008929-RA	11
AGAP008948-RA	11
AGAP009415-RB	11
AGAP009738-RA	11
AGAP010206-RA	11
AGAP010336-RA	11
AGAP011045-RA	11
AGAP011337-RA	11
AGAP011839-RA	11
AGAP011892-RA	11
AGAP000099-RA	11
AGAP000440-RA	11
AGAP000470-RA	11
AGAP000780-RA	11
AGAP000784-RA	11
AGAP002127-RA	11

AGAP002239-RA	11
AGAP002534-RA	11
AGAP002597-RA	11
AGAP002940-RA	11
AGAP003485-RA	11
AGAP003600-RA	11
AGAP003648-RA	11
AGAP005540-RA	11
AGAP006244-RA	11
AGAP007244-RA	11
AGAP007727-RA	11
AGAP008016-RA	11
AGAP008099-RA	11
AGAP008318-RA	11
AGAP008974-RA	11
AGAP009193-RA	11
AGAP009289-RA	11
AGAP009667-RA	11
AGAP009811-RA	11
AGAP010346-RA	11
AGAP011374-RA	11
AGAP011767-RA	11
AGAP011938-RA	11
AGAP028141-RA	11
AGAP000900-RA	11
AGAP001300-RA	11
AGAP001311-RA	11
AGAP001460-RA	11
AGAP003142-RA	11
AGAP005155-RA	11
AGAP005335-RA	11
AGAP005418-RA	11
AGAP006625-RA	11
AGAP006894-RA	11
AGAP007523-RB	11
AGAP007605-RA	11
AGAP008195-RA	11
AGAP009867-RA	11
AGAP010398-RA	11
AGAP010613-RA	11
AGAP010770-RA	11
AGAP011115-RA	11
AGAP004884-RA	11
AGAP004906-RA	11
AGAP005916-RA	11
AGAP007996-RB	11
AGAP009546-RA	11
AGAP010624-RA	11
AGAP010733-RA	11
AGAP010821-RA	11
AGAP011089-RA	11
AGAP011202-RA	11
AGAP011715-RA	11
AGAP012258-RA	11

AGAP000950-RA	11
AGAP000975-RA	11
AGAP001677-RA	11
AGAP003359-RA	11
AGAP003652-RB	11
AGAP003680-RA	11
AGAP003720-RA	11
AGAP004704-RA	11
AGAP005820-RA	11
AGAP009134-RA	11
AGAP010210-RA	11
AGAP010427-RA	11
AGAP011584-RA	11
AGAP011851-RA	11
AGAP000699-RB	11
AGAP000763-RA	11
AGAP000868-RA	11
AGAP002416-RA	11
AGAP002665-RA	11
AGAP002970-RA	11
AGAP003344-RA	11
AGAP004483-RA	11
AGAP005250-RA	11
AGAP005380-RA	11
AGAP006117-RA	11
AGAP006511-RA	11
AGAP006994-RA	11
AGAP007256-RA	11
AGAP008105-RA	11
AGAP008327-RA	11
AGAP008474-RA	11
AGAP008598-RA	11
AGAP008756-RA	11
AGAP011183-RA	11
AGAP028636-RA	11
AGAP000913-RA	11
AGAP001081-RA	11
AGAP002862-RA	11
AGAP002935-RA	11
AGAP003232-RA	11
AGAP004068-RA	11
AGAP005635-RA	11
AGAP005937-RA	11
AGAP006573-RA	11
AGAP006747-RA	11
AGAP007169-RB	11
AGAP007391-RA	11
AGAP008577-RA	11
AGAP008718-RA	11
AGAP009201-RA	11
AGAP011384-RA	11
AGAP001148-RB	11
AGAP001982-RA	11
AGAP002337-RA	11

AGAP005228-RA	11
AGAP005933-RA	11
AGAP006264-RB	11
AGAP010462-RA	11
AGAP012590-RA	11
AGAP003987-RA	11
AGAP009074-RA	11
AGAP002892-RA	11
AGAP007317-RA	11
AGAP007480-RA	11
AGAP010318-RA	11
AGAP000909-RA	11
AGAP001369-RA	11
AGAP001464-RA	11
AGAP001512-RA	11
AGAP004227-RA	11
AGAP005110-RA	11
AGAP006132-RA	11
AGAP008006-RA	11
AGAP009949-RA	11
AGAP002513-RA	11
AGAP004610-RA	11
AGAP000220-RA	11
AGAP000985-RA	11
AGAP002219-RA	11
AGAP008983-RA	11
AGAP009970-RA	11
AGAP009985-RA	11
AGAP000273-RA	11
AGAP000457-RB	11
AGAP001523-RA	11
AGAP001749-RA	11
AGAP003251-RA	11
AGAP009152-RA	11
AGAP011162-RA	11
AGAP028499-RA	11
AGAP001903-RA	11
AGAP002175-RA	11
AGAP003051-RA	11
AGAP004855-RA	11
AGAP009834-RA	11
AGAP010160-RA	11
AGAP008883-RA	11
AGAP004487-RA	11
AGAP009862-RA	12
AGAP028012-RA	12
AGAP000725-RA	12
AGAP003737-RA	12
AGAP005144-RA	12
AGAP005472-RA	12
AGAP006599-RA	12
AGAP006810-RA	12
AGAP006889-RA	12
AGAP006941-RA	12

AGAP007068-RA	12
AGAP007114-RA	12
AGAP007128-RA	12
AGAP007388-RA	12
AGAP007581-RA	12
AGAP007617-RA	12
AGAP009004-RA	12
AGAP009307-RA	12
AGAP009488-RA	12
AGAP009943-RA	12
AGAP010414-RA	12
AGAP010702-RA	12
AGAP010737-RA	12
AGAP010958-RA	12
AGAP011255-RA	12
AGAP011394-RA	12
AGAP011403-RA	12
AGAP011449-RA	12
AGAP011626-RA	12
AGAP011678-RA	12
AGAP012193-RA	12
AGAP012240-RA	12
AGAP012306-RA	12
AGAP012408-RA	12
AGAP012955-RA	12
AGAP013499-RA	12
AGAP013527-RA	12
AGAP028623-RA	12
AGAP028634-RA	12
AGAP000334-RB	12
AGAP000634-RA	12
AGAP001261-RA	12
AGAP001324-RA	12
AGAP001406-RA	12
AGAP001428-RA	12
AGAP001513-RA	12
AGAP001521-RA	12
AGAP001586-RA	12
AGAP001640-RA	12
AGAP001688-RA	12
AGAP001700-RA	12
AGAP001733-RA	12
AGAP001736-RA	12
AGAP001788-RA	12
AGAP001882-RA	12
AGAP001895-RA	12
AGAP001954-RA	12
AGAP001961-RA	12
AGAP002003-RA	12
AGAP002014-RA	12
AGAP002022-RA	12
AGAP002144-RA	12
AGAP002153-RA	12
AGAP002177-RA	12

AGAP002223-RA	12
AGAP002242-RA	12
AGAP002261-RA	12
AGAP002287-RA	12
AGAP002313-RA	12
AGAP002348-RA	12
AGAP002384-RB	12
AGAP002404-RA	12
AGAP002422-RA	12
AGAP002533-RA	12
AGAP002535-RA	12
AGAP002541-RC	12
AGAP002618-RA	12
AGAP002656-RA	12
AGAP002682-RB	12
AGAP002683-RA	12
AGAP002692-RA	12
AGAP002720-RA	12
AGAP002731-RA	12
AGAP002734-RA	12
AGAP002840-RA	12
AGAP002895-RA	12
AGAP002932-RA	12
AGAP002988-RA	12
AGAP003004-RA	12
AGAP003015-RA	12
AGAP003019-RA	12
AGAP003033-RA	12
AGAP003064-RA	12
AGAP003156-RA	12
AGAP003213-RA	12
AGAP003220-RA	12
AGAP003273-RA	12
AGAP003279-RA	12
AGAP003294-RA	12
AGAP003356-RA	12
AGAP003418-RA	12
AGAP003423-RA	12
AGAP003490-RA	12
AGAP003746-RA	12
AGAP003758-RA	12
AGAP003798-RA	12
AGAP003866-RA	12
AGAP003870-RA	12
AGAP003872-RA	12
AGAP003882-RB	12
AGAP003902-RA	12
AGAP003980-RA	12
AGAP003981-RA	12
AGAP003984-RA	12
AGAP003988-RA	12
AGAP004023-RA	12
AGAP004062-RA	12
AGAP004095-RA	12

AGAP004140-RA	12
AGAP004152-RA	12
AGAP004178-RA	12
AGAP004241-RA	12
AGAP004269-RA	12
AGAP004321-RA	12
AGAP004324-RA	12
AGAP004338-RA	12
AGAP004358-RA	12
AGAP004375-RA	12
AGAP004414-RA	12
AGAP004499-RB	12
AGAP004516-RA	12
AGAP004530-RA	12
AGAP004623-RA	12
AGAP004638-RA	12
AGAP004656-RA	12
AGAP004931-RA	12
AGAP005007-RA	12
AGAP005033-RA	12
AGAP005035-RA	12
AGAP005049-RA	12
AGAP005161-RA	12
AGAP005211-RA	12
AGAP005226-RA	12
AGAP005259-RA	12
AGAP005297-RA	12
AGAP005298-RA	12
AGAP005325-RA	12
AGAP005353-RA	12
AGAP005392-RA	12
AGAP005429-RA	12
AGAP005438-RA	12
AGAP005476-RA	12
AGAP005512-RA	12
AGAP005538-RA	12
AGAP005557-RA	12
AGAP005582-RA	12
AGAP005731-RA	12
AGAP005757-RA	12
AGAP005761-RA	12
AGAP005778-RA	12
AGAP005811-RA	12
AGAP005812-RA	12
AGAP005817-RA	12
AGAP005849-RA	12
AGAP005857-RA	12
AGAP005886-RA	12
AGAP005906-RA	12
AGAP005909-RA	12
AGAP005928-RA	12
AGAP005936-RA	12
AGAP005980-RA	12
AGAP006056-RA	12

AGAP006062-RA	12
AGAP006138-RA	12
AGAP006142-RA	12
AGAP006328-RA	12
AGAP006384-RA	12
AGAP006412-RA	12
AGAP006451-RA	12
AGAP006594-RA	12
AGAP006598-RA	12
AGAP006620-RA	12
AGAP006632-RA	12
AGAP006761-RA	12
AGAP006798-RA	12
AGAP006811-RA	12
AGAP007029-RA	12
AGAP007045-RA	12
AGAP007102-RA	12
AGAP007147-RA	12
AGAP007314-RA	12
AGAP007324-RA	12
AGAP007336-RA	12
AGAP007392-RA	12
AGAP007395-RA	12
AGAP007402-RA	12
AGAP007413-RA	12
AGAP007482-RA	12
AGAP007578-RA	12
AGAP007625-RA	12
AGAP007646-RA	12
AGAP007660-RA	12
AGAP007661-RA	12
AGAP007704-RA	12
AGAP007731-RA	12
AGAP007801-RA	12
AGAP007805-RA	12
AGAP007868-RA	12
AGAP007897-RA	12
AGAP007968-RA	12
AGAP007971-RA	12
AGAP007999-RA	12
AGAP008037-RA	12
AGAP008050-RA	12
AGAP008090-RA	12
AGAP008111-RA	12
AGAP008166-RA	12
AGAP008237-RA	12
AGAP008239-RA	12
AGAP008325-RA	12
AGAP008381-RA	12
AGAP008483-RA	12
AGAP008490-RA	12
AGAP008509-RA	12
AGAP008528-RA	12
AGAP008625-RB	12

AGAP008658-RA	12
AGAP008697-RA	12
AGAP008728-RA	12
AGAP008849-RA	12
AGAP008862-RA	12
AGAP008864-RA	12
AGAP008897-RA	12
AGAP008928-RA	12
AGAP008939-RA	12
AGAP008941-RA	12
AGAP008942-RB	12
AGAP008965-RA	12
AGAP008982-RA	12
AGAP008991-RA	12
AGAP009010-RA	12
AGAP009116-RA	12
AGAP009128-RA	12
AGAP009221-RA	12
AGAP009259-RA	12
AGAP009532-RA	12
AGAP009539-RA	12
AGAP009565-RA	12
AGAP009587-RA	12
AGAP009588-RA	12
AGAP009649-RA	12
AGAP009665-RA	12
AGAP009739-RA	12
AGAP009743-RA	12
AGAP009781-RA	12
AGAP009782-RA	12
AGAP009810-RA	12
AGAP009832-RA	12
AGAP009846-RA	12
AGAP009941-RA	12
AGAP010020-RA	12
AGAP010071-RA	12
AGAP010081-RA	12
AGAP010151-RA	12
AGAP010192-RA	12
AGAP010198-RA	12
AGAP010404-RA	12
AGAP010453-RA	12
AGAP010537-RA	12
AGAP010599-RA	12
AGAP010690-RA	12
AGAP010717-RA	12
AGAP010729-RA	12
AGAP010754-RA	12
AGAP010793-RA	12
AGAP010802-RA	12
AGAP010881-RA	12
AGAP010960-RA	12
AGAP010982-RA	12
AGAP011155-RA	12

AGAP011179-RA	12
AGAP011274-RA	12
AGAP011344-RA	12
AGAP011395-RA	12
AGAP011404-RA	12
AGAP011425-RA	12
AGAP011454-RA	12
AGAP011455-RA	12
AGAP011520-RA	12
AGAP011547-RA	12
AGAP011559-RA	12
AGAP011756-RA	12
AGAP011770-RA	12
AGAP011796-RA	12
AGAP011804-RA	12
AGAP011835-RA	12
AGAP011850-RA	12
AGAP011881-RA	12
AGAP012018-RA	12
AGAP012152-RA	12
AGAP012208-RA	12
AGAP012218-RA	12
AGAP012371-RA	12
AGAP012372-RA	12
AGAP012971-RA	12
AGAP013044-RA	12
AGAP013177-RA	12
AGAP013386-RA	12
AGAP013467-RA	12
AGAP028565-RA	12
AGAP000010-RA	12
AGAP000020-RA	12
AGAP000116-RA	12
AGAP000319-RA	12
AGAP000341-RA	12
AGAP000429-RA	12
AGAP000469-RB	12
AGAP000544-RA	12
AGAP000602-RA	12
AGAP000649-RA	12
AGAP000754-RA	12
AGAP000856-RA	12
AGAP000887-RA	12
AGAP000939-RA	12
AGAP000942-RB	12
AGAP000973-RA	12
AGAP000983-RA	12
AGAP001030-RA	12
AGAP001104-RA	12
AGAP001156-RA	12
AGAP001215-RB	12
AGAP001227-RA	12
AGAP001242-RA	12
AGAP001292-RA	12

AGAP001316-RA	12
AGAP001338-RA	12
AGAP001347-RA	12
AGAP001356-RA	12
AGAP001371-RA	12
AGAP001396-RA	12
AGAP001432-RA	12
AGAP001555-RA	12
AGAP001559-RA	12
AGAP001573-RA	12
AGAP001583-RA	12
AGAP001627-RA	12
AGAP001654-RA	12
AGAP001726-RA	12
AGAP001908-RA	12
AGAP001923-RA	12
AGAP002050-RA	12
AGAP002066-RA	12
AGAP002073-RA	12
AGAP002077-RA	12
AGAP002091-RA	12
AGAP002112-RA	12
AGAP002214-RA	12
AGAP002283-RA	12
AGAP002322-RA	12
AGAP002327-RA	12
AGAP002383-RA	12
AGAP002388-RA	12
AGAP002475-RA	12
AGAP002546-RA	12
AGAP002552-RA	12
AGAP002554-RA	12
AGAP002616-RA	12
AGAP002664-RA	12
AGAP002684-RB	12
AGAP002687-RA	12
AGAP002791-RA	12
AGAP002805-RA	12
AGAP002869-RA	12
AGAP002991-RA	12
AGAP003094-RA	12
AGAP003159-RA	12
AGAP003170-RA	12
AGAP003262-RA	12
AGAP003353-RA	12
AGAP003363-RA	12
AGAP003404-RA	12
AGAP003432-RA	12
AGAP003454-RA	12
AGAP003459-RA	12
AGAP003516-RA	12
AGAP003522-RA	12
AGAP003590-RA	12
AGAP003616-RA	12

AGAP003645-RA	12
AGAP003676-RA	12
AGAP003681-RA	12
AGAP003723-RA	12
AGAP003738-RA	12
AGAP003740-RA	12
AGAP003792-RA	12
AGAP003806-RA	12
AGAP003999-RA	12
AGAP004177-RA	12
AGAP004215-RA	12
AGAP004254-RA	12
AGAP004311-RA	12
AGAP004401-RA	12
AGAP004467-RA	12
AGAP004509-RB	12
AGAP004557-RA	12
AGAP004614-RA	12
AGAP004736-RA	12
AGAP004955-RA	12
AGAP005036-RA	12
AGAP005048-RA	12
AGAP005056-RA	12
AGAP005133-RA	12
AGAP005198-RA	12
AGAP005223-RA	12
AGAP005242-RA	12
AGAP005262-RA	12
AGAP005290-RA	12
AGAP005307-RA	12
AGAP005343-RA	12
AGAP005347-RA	12
AGAP005394-RA	12
AGAP005409-RA	12
AGAP005410-RC	12
AGAP005477-RA	12
AGAP005515-RA	12
AGAP005605-RA	12
AGAP005623-RA	12
AGAP005653-RA	12
AGAP005699-RA	12
AGAP005905-RA	12
AGAP005955-RA	12
AGAP006046-RA	12
AGAP006049-RA	12
AGAP006092-RA	12
AGAP006162-RA	12
AGAP006263-RA	12
AGAP006438-RA	12
AGAP006605-RA	12
AGAP006623-RA	12
AGAP006975-RA	12
AGAP007026-RA	12
AGAP007064-RA	12

AGAP007140-RA	12
AGAP007301-RA	12
AGAP007311-RA	12
AGAP007348-RA	12
AGAP007495-RA	12
AGAP007516-RA	12
AGAP007547-RA	12
AGAP007568-RA	12
AGAP007826-RA	12
AGAP007827-RB	12
AGAP007834-RA	12
AGAP007894-RA	12
AGAP007896-RA	12
AGAP007910-RA	12
AGAP007953-RA	12
AGAP008084-RA	12
AGAP008110-RA	12
AGAP008123-RA	12
AGAP008417-RA	12
AGAP008504-RA	12
AGAP008621-RA	12
AGAP008832-RA	12
AGAP008857-RA	12
AGAP008881-RA	12
AGAP008887-RA	12
AGAP008923-RA	12
AGAP008954-RA	12
AGAP009171-RA	12
AGAP009185-RA	12
AGAP009279-RA	12
AGAP009487-RA	12
AGAP009506-RA	12
AGAP009585-RA	12
AGAP009608-RA	12
AGAP009644-RA	12
AGAP009674-RA	12
AGAP009681-RA	12
AGAP009791-RA	12
AGAP009905-RA	12
AGAP009909-RA	12
AGAP009956-RA	12
AGAP010134-RA	12
AGAP010215-RA	12
AGAP010237-RA	12
AGAP010577-RA	12
AGAP010671-RA	12
AGAP010698-RA	12
AGAP011186-RA	12
AGAP011249-RA	12
AGAP011401-RA	12
AGAP011623-RA	12
AGAP011693-RA	12
AGAP011858-RA	12
AGAP011879-RA	12

AGAP012264-RA	12
AGAP012359-RB	12
AGAP012393-RA	12
AGAP012444-RA	12
AGAP012663-RA	12
AGAP013088-RA	12
AGAP013380-RA	12
AGAP013385-RA	12
AGAP013392-RA	12
AGAP000105-RA	12
AGAP000143-RA	12
AGAP000201-RA	12
AGAP000250-RA	12
AGAP000271-RA	12
AGAP000330-RA	12
AGAP000365-RA	12
AGAP000492-RA	12
AGAP000513-RB	12
AGAP000659-RA	12
AGAP000668-RA	12
AGAP000768-RA	12
AGAP000850-RA	12
AGAP000884-RB	12
AGAP001269-RB	12
AGAP001442-RA	12
AGAP001590-RA	12
AGAP001648-RA	12
AGAP001769-RD	12
AGAP001798-RA	12
AGAP001871-RA	12
AGAP001879-RA	12
AGAP001907-RA	12
AGAP001938-RA	12
AGAP002195-RB	12
AGAP002221-RA	12
AGAP002420-RA	12
AGAP002666-RA	12
AGAP002710-RA	12
AGAP002822-RA	12
AGAP002966-RA	12
AGAP003041-RA	12
AGAP003203-RA	12
AGAP003483-RA	12
AGAP003542-RA	12
AGAP004099-RA	12
AGAP004164-RC	12
AGAP004265-RA	12
AGAP004291-RA	12
AGAP005249-RA	12
AGAP005656-RA	12
AGAP005683-RA	12
AGAP005769-RA	12
AGAP005920-RA	12
AGAP006061-RA	12

AGAP006392-RA	12
AGAP006624-RA	12
AGAP007076-RA	12
AGAP007096-RA	12
AGAP007145-RA	12
AGAP007356-RA	12
AGAP007730-RA	12
AGAP007895-RA	12
AGAP008125-RA	12
AGAP008242-RA	12
AGAP008488-RA	12
AGAP008780-RA	12
AGAP008904-RA	12
AGAP009114-RA	12
AGAP009141-RA	12
AGAP009786-RA	12
AGAP009939-RA	12
AGAP010152-RA	12
AGAP010178-RA	12
AGAP010345-RA	12
AGAP010389-RA	12
AGAP010550-RA	12
AGAP010857-RA	12
AGAP011178-RA	12
AGAP011252-RA	12
AGAP011326-RA	12
AGAP011491-RA	12
AGAP011750-RA	12
AGAP028430-RA	12
AGAP000033-RA	12
AGAP000223-RA	12
AGAP000499-RA	12
AGAP000769-RB	12
AGAP000933-RB	12
AGAP000981-RA	12
AGAP001011-RA	12
AGAP001400-RA	12
AGAP001535-RA	12
AGAP001570-RA	12
AGAP001916-RA	12
AGAP001917-RA	12
AGAP001974-RA	12
AGAP002426-RA	12
AGAP003093-RA	12
AGAP003593-RA	12
AGAP004292-RA	12
AGAP004909-RA	12
AGAP004952-RA	12
AGAP005102-RA	12
AGAP005270-RA	12
AGAP005366-RA	12
AGAP005541-RA	12
AGAP005583-RA	12
AGAP005780-RA	12

AGAP005853-RA	12
AGAP005882-RA	12
AGAP006082-RA	12
AGAP006120-RA	12
AGAP006235-RA	12
AGAP006629-RA	12
AGAP007595-RA	12
AGAP007686-RA	12
AGAP008092-RA	12
AGAP008190-RA	12
AGAP008707-RA	12
AGAP009549-RA	12
AGAP009579-RA	12
AGAP009888-RA	12
AGAP009980-RA	12
AGAP010019-RA	12
AGAP010625-RA	12
AGAP010757-RA	12
AGAP011383-RA	12
AGAP011488-RA	12
AGAP011522-RA	12
AGAP011533-RA	12
AGAP012440-RA	12
AGAP028502-RA	12
AGAP000158-RA	12
AGAP000391-RA	12
AGAP001362-RA	12
AGAP001729-RA	12
AGAP001909-RA	12
AGAP002246-RA	12
AGAP002570-RA	12
AGAP002801-RA	12
AGAP004608-RA	12
AGAP004903-RA	12
AGAP005115-RA	12
AGAP005631-RA	12
AGAP005738-RA	12
AGAP005990-RA	12
AGAP006600-RA	12
AGAP007509-RA	12
AGAP007739-RA	12
AGAP007819-RA	12
AGAP008160-RA	12
AGAP008185-RA	12
AGAP008522-RA	12
AGAP009161-RA	12
AGAP009178-RA	12
AGAP009650-RA	12
AGAP009798-RA	12
AGAP009931-RA	12
AGAP010538-RA	12
AGAP010646-RA	12
AGAP011568-RA	12
AGAP012417-RA	12

AGAP012991-RA	12
AGAP000454-RA	12
AGAP000480-RA	12
AGAP001561-RB	12
AGAP001589-RA	12
AGAP001629-RA	12
AGAP001707-RA	12
AGAP002717-RA	12
AGAP002780-RA	12
AGAP003268-RA	12
AGAP003434-RA	12
AGAP004020-RA	12
AGAP004423-RA	12
AGAP005018-RA	12
AGAP005020-RA	12
AGAP005615-RA	12
AGAP005753-RA	12
AGAP006355-RA	12
AGAP007054-RA	12
AGAP007115-RA	12
AGAP007212-RA	12
AGAP007531-RA	12
AGAP007773-RA	12
AGAP008167-RA	12
AGAP008362-RA	12
AGAP008910-RA	12
AGAP010034-RA	12
AGAP010167-RB	12
AGAP010380-RA	12
AGAP011205-RA	12
AGAP011753-RA	12
AGAP012301-RA	12
AGAP012379-RA	12
AGAP012381-RA	12
AGAP012957-RA	12
AGAP000040-RA	12
AGAP000532-RB	12
AGAP000563-RA	12
AGAP001286-RA	12
AGAP001450-RA	12
AGAP001644-RB	12
AGAP001792-RA	12
AGAP002065-RA	12
AGAP002151-RA	12
AGAP002154-RB	12
AGAP002605-RA	12
AGAP002663-RA	12
AGAP003035-RA	12
AGAP004240-RA	12
AGAP004419-RA	12
AGAP005136-RA	12
AGAP005648-RA	12
AGAP006479-RA	12
AGAP006528-RA	12

AGAP006768-RA	12
AGAP006960-RA	12
AGAP007176-RA	12
AGAP007377-RA	12
AGAP007655-RA	12
AGAP008048-RA	12
AGAP008300-RA	12
AGAP010587-RA	12
AGAP010833-RA	12
AGAP010834-RA	12
AGAP010959-RA	12
AGAP011070-RA	12
AGAP011984-RA	12
AGAP012922-RA	12
AGAP000110-RA	12
AGAP000269-RA	12
AGAP000549-RA	12
AGAP001234-RA	12
AGAP001565-RA	12
AGAP001752-RA	12
AGAP001757-RA	12
AGAP002427-RA	12
AGAP002512-RA	12
AGAP003029-RA	12
AGAP003562-RA	12
AGAP004229-RA	12
AGAP004345-RA	12
AGAP004361-RA	12
AGAP004785-RA	12
AGAP005331-RA	12
AGAP005526-RA	12
AGAP005553-RA	12
AGAP005922-RA	12
AGAP005973-RA	12
AGAP006509-RA	12
AGAP006580-RA	12
AGAP007335-RA	12
AGAP008124-RA	12
AGAP008687-RA	12
AGAP008720-RA	12
AGAP009688-RA	12
AGAP010042-RA	12
AGAP010274-RA	12
AGAP010711-RA	12
AGAP010782-RA	12
AGAP011519-RA	12
AGAP011932-RA	12
AGAP012336-RA	12
AGAP001426-RA	12
AGAP001686-RA	12
AGAP002224-RA	12
AGAP002258-RB	12
AGAP003073-RB	12
AGAP003230-RA	12

AGAP003368-RA	12
AGAP003820-RA	12
AGAP003906-RA	12
AGAP003974-RA	12
AGAP004409-RA	12
AGAP004729-RA	12
AGAP004781-RA	12
AGAP004905-RA	12
AGAP005575-RA	12
AGAP005584-RA	12
AGAP005616-RA	12
AGAP006251-RA	12
AGAP006281-RA	12
AGAP006633-RA	12
AGAP006880-RA	12
AGAP007080-RA	12
AGAP007507-RA	12
AGAP007529-RA	12
AGAP007772-RA	12
AGAP007932-RA	12
AGAP007965-RA	12
AGAP008209-RA	12
AGAP008717-RA	12
AGAP008882-RA	12
AGAP008900-RA	12
AGAP009133-RA	12
AGAP011198-RA	12
AGAP011492-RA	12
AGAP011541-RA	12
AGAP012289-RA	12
AGAP013221-RA	12
AGAP013463-RA	12
AGAP000119-RA	12
AGAP000394-RA	12
AGAP000529-RA	12
AGAP000536-RA	12
AGAP000857-RA	12
AGAP001167-RA	12
AGAP001401-RA	12
AGAP002832-RA	12
AGAP002929-RA	12
AGAP002959-RA	12
AGAP003182-RA	12
AGAP003187-RA	12
AGAP003252-RA	12
AGAP003585-RA	12
AGAP003970-RA	12
AGAP004847-RA	12
AGAP005537-RA	12
AGAP005871-RA	12
AGAP006239-RA	12
AGAP006770-RA	12
AGAP006780-RA	12
AGAP007419-RA	12

AGAP008127-RA	12
AGAP008159-RA	12
AGAP008336-RA	12
AGAP008397-RA	12
AGAP008614-RA	12
AGAP008757-RA	12
AGAP008828-RA	12
AGAP008876-RA	12
AGAP012083-RA	12
AGAP028125-RA	12
AGAP000436-RA	12
AGAP000545-RA	12
AGAP000967-RA	12
AGAP001310-RA	12
AGAP001386-RA	12
AGAP001936-RA	12
AGAP001955-RB	12
AGAP002142-RA	12
AGAP002425-RA	12
AGAP002712-RA	12
AGAP003146-RA	12
AGAP003158-RA	12
AGAP003506-RA	12
AGAP004071-RB	12
AGAP004120-RA	12
AGAP004480-RA	12
AGAP004523-RA	12
AGAP005640-RA	12
AGAP006130-RA	12
AGAP007117-RA	12
AGAP007626-RA	12
AGAP007983-RC	12
AGAP008875-RA	12
AGAP009400-RA	12
AGAP009838-RA	12
AGAP010176-RA	12
AGAP010629-RA	12
AGAP010712-RA	12
AGAP011125-RA	12
AGAP011686-RA	12
AGAP012013-RA	12
AGAP012066-RA	12
AGAP012144-RA	12
AGAP013008-RA	12
AGAP001233-RA	12
AGAP001492-RA	12
AGAP001656-RA	12
AGAP001888-RA	12
AGAP001943-RA	12
AGAP002012-RA	12
AGAP002048-RB	12
AGAP002600-RA	12
AGAP003943-RA	12
AGAP004839-RA	12

AGAP004954-RA	12
AGAP005624-RB	12
AGAP006608-RA	12
AGAP006714-RA	12
AGAP007390-RA	12
AGAP008330-RA	12
AGAP008407-RA	12
AGAP012116-RA	12
AGAP000861-RA	12
AGAP001150-RA	12
AGAP001737-RA	12
AGAP002567-RA	12
AGAP002680-RA	12
AGAP002782-RA	12
AGAP002875-RA	12
AGAP003171-RA	12
AGAP005119-RA	12
AGAP005428-RA	12
AGAP005654-RA	12
AGAP006789-RB	12
AGAP010141-RA	12
AGAP010419-RA	12
AGAP000261-RA	12
AGAP000601-RB	12
AGAP002406-RA	12
AGAP002829-RA	12
AGAP003464-RA	12
AGAP003745-RA	12
AGAP004820-RA	12
AGAP005544-RA	12
AGAP009304-RA	12
AGAP009439-RA	12
AGAP012657-RA	12
AGAP028133-RA	12
AGAP001278-RA	12
AGAP001878-RA	12
AGAP002174-RA	12
AGAP003884-RB	12
AGAP004087-RA	12
AGAP004793-RA	12
AGAP007313-RA	12
AGAP009675-RA	12
AGAP010135-RA	12
AGAP001297-RA	12
AGAP001402-RA	12
AGAP002409-RA	12
AGAP002424-RA	12
AGAP005424-RA	12
AGAP007379-RA	12
AGAP007808-RB	12
AGAP007835-RA	12
AGAP008148-RA	12
AGAP011248-RA	12
AGAP012101-RA	12

AGAP000428-RA	12
AGAP001139-RA	12
AGAP003178-RA	12
AGAP003936-RA	12
AGAP003976-RA	12
AGAP004429-RA	12
AGAP006815-RA	12
AGAP008877-RA	12
AGAP002527-RA	12
AGAP004112-RA	12
AGAP005252-RA	12
AGAP008660-RA	12
AGAP001255-RA	12
AGAP001603-RA	12
AGAP002985-RA	12
AGAP003595-RA	12
AGAP005323-RB	12
AGAP007043-RB	12
AGAP010308-RA	12
AGAP010728-RA	12
AGAP011745-RA	12
AGAP000163-RA	12
AGAP003727-RA	12
AGAP008041-RA	12
AGAP011142-RA	12
AGAP003476-RA	12
AGAP010491-RA	12
AGAP002269-RA	12
AGAP003322-RA	12
AGAP008303-RA	12
AGAP009979-RA	12
AGAP000127-RA	12
AGAP002094-RA	12
AGAP003028-RA	12
AGAP003726-RA	12
AGAP004938-RA	12
AGAP001367-RA	12
AGAP005227-RA	12
AGAP008131-RA	12
AGAP000908-RA	12
AGAP005169-RA	13
AGAP007979-RA	13
AGAP007989-RA	13
AGAP008426-RA	13
AGAP009137-RA	13
AGAP009550-RA	13
AGAP010014-RA	13
AGAP010554-RA	13
AGAP011590-RA	13
AGAP013343-RA	13
AGAP001241-RA	13
AGAP001429-RA	13
AGAP002543-RA	13
AGAP003547-RB	13

AGAP003855-RA	13
AGAP005125-RA	13
AGAP005158-RA	13
AGAP005215-RA	13
AGAP005349-RA	13
AGAP005417-RA	13
AGAP005634-RA	13
AGAP005804-RA	13
AGAP005842-RA	13
AGAP006241-RA	13
AGAP006349-RA	13
AGAP006785-RA	13
AGAP007052-RA	13
AGAP007126-RA	13
AGAP007502-RA	13
AGAP007662-RA	13
AGAP007679-RA	13
AGAP007706-RA	13
AGAP007798-RA	13
AGAP008005-RA	13
AGAP008022-RA	13
AGAP008627-RA	13
AGAP008629-RA	13
AGAP008671-RA	13
AGAP008672-RA	13
AGAP009047-RA	13
AGAP009225-RA	13
AGAP009274-RB	13
AGAP009573-RA	13
AGAP009586-RA	13
AGAP009655-RA	13
AGAP009735-RA	13
AGAP009881-RA	13
AGAP009996-RA	13
AGAP010043-RA	13
AGAP010055-RA	13
AGAP010180-RA	13
AGAP010219-RA	13
AGAP010418-RA	13
AGAP010699-RA	13
AGAP010715-RA	13
AGAP010786-RA	13
AGAP010787-RA	13
AGAP010788-RA	13
AGAP010836-RA	13
AGAP011103-RA	13
AGAP011116-RA	13
AGAP011246-RA	13
AGAP011302-RA	13
AGAP011340-RA	13
AGAP011407-RB	13
AGAP011409-RA	13
AGAP011442-RA	13
AGAP011485-RA	13

AGAP011496-RA	13
AGAP011643-RA	13
AGAP011712-RA	13
AGAP011713-RA	13
AGAP011760-RA	13
AGAP011819-RA	13
AGAP011900-RA	13
AGAP012003-RA	13
AGAP012099-RA	13
AGAP012119-RA	13
AGAP012122-RA	13
AGAP012160-RA	13
AGAP012165-RA	13
AGAP012245-RA	13
AGAP012286-RA	13
AGAP012419-RA	13
AGAP012768-RA	13
AGAP012951-RA	13
AGAP027998-RA	13
AGAP028019-RA	13
AGAP028040-RA	13
AGAP028145-RA	13
AGAP028745-RA	13
AGAP000072-RA	13
AGAP000130-RA	13
AGAP000154-RA	13
AGAP000240-RA	13
AGAP000242-RA	13
AGAP000390-RA	13
AGAP000398-RA	13
AGAP000535-RA	13
AGAP000732-RA	13
AGAP000751-RA	13
AGAP000907-RA	13
AGAP001019-RA	13
AGAP001135-RA	13
AGAP001294-RA	13
AGAP001326-RA	13
AGAP001333-RA	13
AGAP001379-RA	13
AGAP001425-RA	13
AGAP001449-RA	13
AGAP001458-RA	13
AGAP001474-RA	13
AGAP001534-RA	13
AGAP001556-RA	13
AGAP001562-RA	13
AGAP001623-RA	13
AGAP001756-RA	13
AGAP001793-RA	13
AGAP001814-RA	13
AGAP001854-RA	13
AGAP001881-RA	13
AGAP001913-RA	13

AGAP001929-RA	13
AGAP001948-RA	13
AGAP001949-RA	13
AGAP001972-RA	13
AGAP002004-RA	13
AGAP002106-RA	13
AGAP002126-RA	13
AGAP002255-RA	13
AGAP002394-RA	13
AGAP002474-RA	13
AGAP002476-RA	13
AGAP002495-RA	13
AGAP002580-RA	13
AGAP002602-RA	13
AGAP002678-RA	13
AGAP002703-RA	13
AGAP002778-RA	13
AGAP002865-RA	13
AGAP002967-RA	13
AGAP003032-RA	13
AGAP003123-RA	13
AGAP003161-RA	13
AGAP003180-RA	13
AGAP003214-RA	13
AGAP003303-RA	13
AGAP003394-RA	13
AGAP003401-RA	13
AGAP003482-RA	13
AGAP003510-RA	13
AGAP003527-RA	13
AGAP003611-RA	13
AGAP003646-RA	13
AGAP003718-RA	13
AGAP003796-RA	13
AGAP003801-RA	13
AGAP003812-RA	13
AGAP003852-RA	13
AGAP003898-RA	13
AGAP003994-RA	13
AGAP004012-RA	13
AGAP004082-RA	13
AGAP004101-RA	13
AGAP004103-RA	13
AGAP004108-RA	13
AGAP004377-RA	13
AGAP004386-RA	13
AGAP004500-RA	13
AGAP004563-RA	13
AGAP004753-RA	13
AGAP004782-RA	13
AGAP004850-RA	13
AGAP004956-RA	13
AGAP005045-RA	13
AGAP005126-RA	13

AGAP005176-RA	13
AGAP005498-RA	13
AGAP005503-RA	13
AGAP005525-RA	13
AGAP005732-RA	13
AGAP005754-RA	13
AGAP006021-RA	13
AGAP006055-RA	13
AGAP006065-RA	13
AGAP006098-RA	13
AGAP006166-RA	13
AGAP006187-RA	13
AGAP006595-RA	13
AGAP006642-RA	13
AGAP006707-RA	13
AGAP006781-RA	13
AGAP006805-RA	13
AGAP006968-RA	13
AGAP006997-RA	13
AGAP007083-RA	13
AGAP007219-RA	13
AGAP007285-RA	13
AGAP007288-RA	13
AGAP007295-RA	13
AGAP007439-RA	13
AGAP007484-RA	13
AGAP007544-RA	13
AGAP007560-RA	13
AGAP007613-RA	13
AGAP007672-RA	13
AGAP007680-RA	13
AGAP007769-RA	13
AGAP007878-RA	13
AGAP008081-RA	13
AGAP008108-RA	13
AGAP008273-RA	13
AGAP008420-RA	13
AGAP008429-RA	13
AGAP008469-RA	13
AGAP008523-RA	13
AGAP008548-RA	13
AGAP008648-RA	13
AGAP008659-RA	13
AGAP008716-RA	13
AGAP008726-RA	13
AGAP008738-RA	13
AGAP008831-RA	13
AGAP008872-RA	13
AGAP009050-RA	13
AGAP009084-RA	13
AGAP009088-RA	13
AGAP009089-RA	13
AGAP009277-RA	13
AGAP009349-RA	13

AGAP009448-RA	13
AGAP009480-RA	13
AGAP009618-RA	13
AGAP009629-RA	13
AGAP009960-RA	13
AGAP009984-RA	13
AGAP010426-RA	13
AGAP010567-RA	13
AGAP010791-RA	13
AGAP010835-RA	13
AGAP010898-RA	13
AGAP010918-RA	13
AGAP010943-RA	13
AGAP011163-RA	13
AGAP011232-RA	13
AGAP011433-RA	13
AGAP011475-RA	13
AGAP011536-RA	13
AGAP011538-RA	13
AGAP011562-RA	13
AGAP011652-RA	13
AGAP011676-RA	13
AGAP011769-RA	13
AGAP011969-RA	13
AGAP012002-RA	13
AGAP012521-RA	13
AGAP013066-RA	13
AGAP013180-RA	13
AGAP013258-RA	13
AGAP013720-RA	13
AGAP028415-RA	13
AGAP028417-RA	13
AGAP028525-RA	13
AGAP028538-RA	13
AGAP028611-RA	13
AGAP028642-RA	13
AGAP028667-RA	13
AGAP028681-RA	13
AGAP028723-RA	13
AGAP000012-RA	13
AGAP000100-RA	13
AGAP000301-RA	13
AGAP000395-RA	13
AGAP000397-RA	13
AGAP000495-RA	13
AGAP000497-RA	13
AGAP001158-RA	13
AGAP001161-RA	13
AGAP001166-RA	13
AGAP001226-RA	13
AGAP001309-RA	13
AGAP001453-RA	13
AGAP001537-RA	13
AGAP001593-RA	13

AGAP001602-RA	13
AGAP001625-RA	13
AGAP001635-RA	13
AGAP001687-RB	13
AGAP001897-RA	13
AGAP002133-RA	13
AGAP002471-RA	13
AGAP002522-RA	13
AGAP002532-RA	13
AGAP002615-RA	13
AGAP002796-RA	13
AGAP002836-RB	13
AGAP002977-RA	13
AGAP002980-RA	13
AGAP003209-RA	13
AGAP003225-RA	13
AGAP003257-RA	13
AGAP003275-RA	13
AGAP003441-RA	13
AGAP003502-RA	13
AGAP003743-RA	13
AGAP003825-RA	13
AGAP004594-RB	13
AGAP004921-RA	13
AGAP005052-RA	13
AGAP005066-RA	13
AGAP005479-RA	13
AGAP005490-RA	13
AGAP005513-RA	13
AGAP005565-RA	13
AGAP005644-RA	13
AGAP005819-RA	13
AGAP006047-RA	13
AGAP006126-RB	13
AGAP006172-RA	13
AGAP006535-RA	13
AGAP006663-RA	13
AGAP007368-RA	13
AGAP007370-RA	13
AGAP007417-RA	13
AGAP007456-RA	13
AGAP007487-RA	13
AGAP007549-RA	13
AGAP007738-RA	13
AGAP008117-RA	13
AGAP008164-RA	13
AGAP008293-RA	13
AGAP008402-RA	13
AGAP008499-RA	13
AGAP008539-RA	13
AGAP008961-RA	13
AGAP009085-RA	13
AGAP009109-RA	13
AGAP009151-RA	13

AGAP009363-RA	13
AGAP009460-RA	13
AGAP009967-RA	13
AGAP009999-RA	13
AGAP010264-RA	13
AGAP010720-RA	13
AGAP011684-RA	13
AGAP011952-RA	13
AGAP012033-RA	13
AGAP012974-RA	13
AGAP013133-RA	13
AGAP013477-RA	13
AGAP013746-RA	13
AGAP027983-RA	13
AGAP028111-RA	13
AGAP028528-RA	13
AGAP028550-RA	13
AGAP028592-RA	13
AGAP028647-RA	13
AGAP000071-RA	13
AGAP000387-RA	13
AGAP000636-RA	13
AGAP000912-RA	13
AGAP000928-RA	13
AGAP001175-RA	13
AGAP001301-RA	13
AGAP001395-RA	13
AGAP001591-RA	13
AGAP001684-RA	13
AGAP001809-RA	13
AGAP001815-RA	13
AGAP001950-RA	13
AGAP002259-RA	13
AGAP002366-RA	13
AGAP002375-RA	13
AGAP002642-RA	13
AGAP002724-RA	13
AGAP002820-RA	13
AGAP003010-RA	13
AGAP003323-RA	13
AGAP004057-RA	13
AGAP004270-RA	13
AGAP004957-RA	13
AGAP005481-RA	13
AGAP006324-RA	13
AGAP006446-RA	13
AGAP007323-RA	13
AGAP007458-RA	13
AGAP007817-RA	13
AGAP008135-RA	13
AGAP008388-RA	13
AGAP008599-RA	13
AGAP008835-RA	13
AGAP008858-RA	13

AGAP009478-RA	13
AGAP010132-RA	13
AGAP010722-RA	13
AGAP011718-RA	13
AGAP012017-RA	13
AGAP012743-RA	13
AGAP013472-RA	13
AGAP028114-RA	13
AGAP001198-RA	13
AGAP001483-RA	13
AGAP001728-RA	13
AGAP002017-RA	13
AGAP002669-RA	13
AGAP002867-RA	13
AGAP003989-RA	13
AGAP004828-RA	13
AGAP005447-RA	13
AGAP006144-RA	13
AGAP007369-RA	13
AGAP007691-RC	13
AGAP007859-RA	13
AGAP008187-RA	13
AGAP008399-RA	13
AGAP008937-RA	13
AGAP009561-RA	13
AGAP009702-RA	13
AGAP010077-RA	13
AGAP010685-RA	13
AGAP011434-RA	13
AGAP013170-RA	13
AGAP028562-RA	13
AGAP008927-RA	13
AGAP028668-RA	13
AGAP000326-RA	13
AGAP000478-RA	13
AGAP005461-RA	13
AGAP006403-RA	13
AGAP009807-RA	13
AGAP010680-RA	13
AGAP001004-RA	13
AGAP003920-RA	13
AGAP007883-RA	13
AGAP011508-RA	13
AGAP012180-RA	13
AGAP028673-RA	13
AGAP001223-RA	13
AGAP001539-RA	13
AGAP003042-RA	13
AGAP004224-RA	13
AGAP001725-RA	13
AGAP003655-RA	13
AGAP005743-RA	13
AGAP009611-RA	13
AGAP028050-RA	13

AGAP028683-RA	13
AGAP002359-RA	13
AGAP003503-RA	13
AGAP003805-RA	13
AGAP004104-RB	13
AGAP004856-RA	13
AGAP005800-RA	13
AGAP006618-RA	13
AGAP006687-RA	13
AGAP007703-RA	13
AGAP008349-RA	13
AGAP010807-RA	13
AGAP000288-RA	13
AGAP001478-RA	13
AGAP005070-RA	13
AGAP006358-RA	13
AGAP007506-RA	13
AGAP009784-RA	13
AGAP010930-RA	13
AGAP010932-RA	13
AGAP000724-RA	13
AGAP001146-RA	13
AGAP005190-RA	13
AGAP005480-RA	13
AGAP008550-RA	13
AGAP009043-RA	13
AGAP010271-RA	13
AGAP010574-RA	13
AGAP012044-RA	13
AGAP012227-RA	13
AGAP001214-RA	13
AGAP002992-RC	13
AGAP003316-RA	13
AGAP005673-RA	13
AGAP007775-RA	13
AGAP008748-RA	13
AGAP009319-RA	13
AGAP010941-RA	13
AGAP003705-RA	13
AGAP008246-RA	13
AGAP002392-RA	13
AGAP003889-RA	13
AGAP005601-RA	13
AGAP012297-RA	13
AGAP006109-RA	13
AGAP011742-RA	13
AGAP008161-RA	13
AGAP009766-RA	13
AGAP002753-RA	13
AGAP003396-RA	13
AGAP012226-RA	13
AGAP003846-RA	13
AGAP011136-RA	13
AGAP000079-RA	14

AGAP000413-RA	14
AGAP000507-RA	14
AGAP000848-RA	14
AGAP002053-RA	14
AGAP002110-RA	14
AGAP002973-RA	14
AGAP003020-RA	14
AGAP003211-RA	14
AGAP003465-RA	14
AGAP003780-RA	14
AGAP003781-RA	14
AGAP003877-RA	14
AGAP003885-RA	14
AGAP004198-RA	14
AGAP004242-RA	14
AGAP004295-RA	14
AGAP004334-RA	14
AGAP004571-RA	14
AGAP004914-RA	14
AGAP006020-RA	14
AGAP006105-RA	14
AGAP006368-RA	14
AGAP006381-RA	14
AGAP006737-RA	14
AGAP006832-RA	14
AGAP006872-RA	14
AGAP006923-RA	14
AGAP007122-RA	14
AGAP007450-RA	14
AGAP007656-RA	14
AGAP007676-RA	14
AGAP007779-RA	14
AGAP008281-RA	14
AGAP008313-RA	14
AGAP008886-RA	14
AGAP009797-RA	14
AGAP010245-RA	14
AGAP011026-RA	14
AGAP011032-RA	14
AGAP011848-RA	14
AGAP012384-RA	14
AGAP013068-RA	14
AGAP013224-RA	14
AGAP013347-RA	14
AGAP013752-RA	14
AGAP028178-RA	14
AGAP028424-RA	14
AGAP000019-RA	14
AGAP000038-RA	14
AGAP000051-RA	14
AGAP000074-RA	14
AGAP000088-RA	14
AGAP000098-RA	14
AGAP000111-RA	14

AGAP000115-RA	14
AGAP000121-RA	14
AGAP000151-RA	14
AGAP000166-RA	14
AGAP000169-RA	14
AGAP000191-RA	14
AGAP000194-RA	14
AGAP000196-RA	14
AGAP000213-RA	14
AGAP000214-RA	14
AGAP000216-RA	14
AGAP000219-RA	14
AGAP000253-RA	14
AGAP000266-RA	14
AGAP000267-RA	14
AGAP000298-RA	14
AGAP000324-RA	14
AGAP000329-RA	14
AGAP000339-RA	14
AGAP000342-RA	14
AGAP000352-RA	14
AGAP000369-RA	14
AGAP000401-RA	14
AGAP000420-RA	14
AGAP000445-RA	14
AGAP000446-RA	14
AGAP000460-RA	14
AGAP000473-RA	14
AGAP000474-RA	14
AGAP000476-RA	14
AGAP000506-RA	14
AGAP000522-RA	14
AGAP000530-RB	14
AGAP000548-RA	14
AGAP000575-RB	14
AGAP000579-RA	14
AGAP000597-RA	14
AGAP000635-RA	14
AGAP000685-RA	14
AGAP000702-RA	14
AGAP000718-RA	14
AGAP000734-RA	14
AGAP000737-RA	14
AGAP000741-RA	14
AGAP000786-RA	14
AGAP000788-RA	14
AGAP000805-RA	14
AGAP000840-RA	14
AGAP000853-RA	14
AGAP000876-RA	14
AGAP000940-RA	14
AGAP000965-RA	14
AGAP000966-RA	14
AGAP000991-RA	14

AGAP000992-RA	14
AGAP001005-RA	14
AGAP001033-RA	14
AGAP001034-RA	14
AGAP001038-RA	14
AGAP001044-RA	14
AGAP001096-RA	14
AGAP001117-RD	14
AGAP001118-RA	14
AGAP001124-RA	14
AGAP001147-RA	14
AGAP001171-RA	14
AGAP001176-RA	14
AGAP001183-RA	14
AGAP001203-RA	14
AGAP001224-RA	14
AGAP001249-RA	14
AGAP001265-RA	14
AGAP001275-RA	14
AGAP001374-RA	14
AGAP001409-RB	14
AGAP001415-RA	14
AGAP001416-RA	14
AGAP001443-RA	14
AGAP001445-RA	14
AGAP001514-RA	14
AGAP001544-RB	14
AGAP001554-RA	14
AGAP001581-RA	14
AGAP001647-RA	14
AGAP001657-RA	14
AGAP001659-RA	14
AGAP001673-RA	14
AGAP001676-RA	14
AGAP001705-RA	14
AGAP001722-RA	14
AGAP001764-RA	14
AGAP001783-RB	14
AGAP001812-RA	14
AGAP001822-RA	14
AGAP001861-RA	14
AGAP001912-RA	14
AGAP001915-RA	14
AGAP001932-RA	14
AGAP001933-RA	14
AGAP001990-RA	14
AGAP002000-RA	14
AGAP002006-RA	14
AGAP002013-RA	14
AGAP002025-RA	14
AGAP002043-RA	14
AGAP002069-RA	14
AGAP002125-RA	14
AGAP002132-RA	14

AGAP002134-RA	14
AGAP002138-RA	14
AGAP002163-RA	14
AGAP002168-RA	14
AGAP002196-RA	14
AGAP002202-RA	14
AGAP002292-RA	14
AGAP002297-RA	14
AGAP002336-RA	14
AGAP002353-RA	14
AGAP002360-RA	14
AGAP002435-RA	14
AGAP002453-RA	14
AGAP002519-RA	14
AGAP002540-RA	14
AGAP002542-RA	14
AGAP002553-RA	14
AGAP002556-RA	14
AGAP002585-RA	14
AGAP002601-RB	14
AGAP002622-RA	14
AGAP002625-RB	14
AGAP002634-RA	14
AGAP002648-RA	14
AGAP002679-RA	14
AGAP002707-RA	14
AGAP002723-RA	14
AGAP002775-RA	14
AGAP002798-RA	14
AGAP002810-RA	14
AGAP002815-RA	14
AGAP002825-RA	14
AGAP002842-RA	14
AGAP002864-RA	14
AGAP002866-RA	14
AGAP002877-RA	14
AGAP002894-RA	14
AGAP002903-RA	14
AGAP002904-RA	14
AGAP002907-RA	14
AGAP002926-RA	14
AGAP002937-RA	14
AGAP002976-RA	14
AGAP003012-RA	14
AGAP003037-RA	14
AGAP003065-RA	14
AGAP003087-RA	14
AGAP003129-RA	14
AGAP003151-RA	14
AGAP003157-RA	14
AGAP003271-RA	14
AGAP003284-RA	14
AGAP003290-RA	14
AGAP003320-RA	14

AGAP003351-RA	14
AGAP003379-RA	14
AGAP003385-RA	14
AGAP003406-RA	14
AGAP003420-RA	14
AGAP003468-RA	14
AGAP003499-RA	14
AGAP003501-RA	14
AGAP003545-RA	14
AGAP003626-RA	14
AGAP003639-RA	14
AGAP003640-RA	14
AGAP003664-RA	14
AGAP003665-RA	14
AGAP003671-RA	14
AGAP003684-RA	14
AGAP003714-RA	14
AGAP003765-RA	14
AGAP003774-RA	14
AGAP003782-RA	14
AGAP003841-RA	14
AGAP003876-RA	14
AGAP003907-RA	14
AGAP003918-RA	14
AGAP003923-RA	14
AGAP003926-RA	14
AGAP004034-RA	14
AGAP004046-RC	14
AGAP004077-RA	14
AGAP004123-RA	14
AGAP004125-RA	14
AGAP004132-RA	14
AGAP004135-RA	14
AGAP004163-RB	14
AGAP004166-RA	14
AGAP004167-RA	14
AGAP004222-RB	14
AGAP004287-RA	14
AGAP004379-RA	14
AGAP004497-RA	14
AGAP004498-RA	14
AGAP004555-RA	14
AGAP004579-RA	14
AGAP004599-RA	14
AGAP004811-RA	14
AGAP004871-RA	14
AGAP004907-RA	14
AGAP004930-RA	14
AGAP004968-RA	14
AGAP004970-RA	14
AGAP004996-RA	14
AGAP005002-RA	14
AGAP005017-RA	14
AGAP005068-RA	14

AGAP005072-RA	14
AGAP005137-RA	14
AGAP005163-RA	14
AGAP005171-RA	14
AGAP005217-RA	14
AGAP005258-RA	14
AGAP005328-RA	14
AGAP005330-RA	14
AGAP005333-RA	14
AGAP005355-RA	14
AGAP005391-RA	14
AGAP005407-RA	14
AGAP005450-RA	14
AGAP005455-RA	14
AGAP005495-RA	14
AGAP005511-RA	14
AGAP005518-RA	14
AGAP005527-RA	14
AGAP005578-RA	14
AGAP005580-RA	14
AGAP005586-RA	14
AGAP005604-RA	14
AGAP005629-RA	14
AGAP005679-RA	14
AGAP005733-RA	14
AGAP005734-RA	14
AGAP005768-RA	14
AGAP005770-RA	14
AGAP005790-RA	14
AGAP005833-RA	14
AGAP005846-RA	14
AGAP005859-RA	14
AGAP005862-RA	14
AGAP005895-RA	14
AGAP005902-RA	14
AGAP005908-RA	14
AGAP005919-RA	14
AGAP005923-RA	14
AGAP005958-RA	14
AGAP005959-RA	14
AGAP005995-RA	14
AGAP006029-RA	14
AGAP006039-RA	14
AGAP006054-RA	14
AGAP006057-RA	14
AGAP006074-RA	14
AGAP006086-RA	14
AGAP006095-RA	14
AGAP006146-RA	14
AGAP006149-RA	14
AGAP006151-RA	14
AGAP006164-RA	14
AGAP006167-RA	14
AGAP006176-RB	14

AGAP006178-RA	14
AGAP006182-RA	14
AGAP006185-RA	14
AGAP006208-RA	14
AGAP006242-RA	14
AGAP006252-RA	14
AGAP006261-RA	14
AGAP006265-RA	14
AGAP006283-RB	14
AGAP006343-RA	14
AGAP006385-RA	14
AGAP006387-RA	14
AGAP006406-RA	14
AGAP006428-RA	14
AGAP006434-RA	14
AGAP006435-RA	14
AGAP006447-RA	14
AGAP006450-RA	14
AGAP006465-RA	14
AGAP006466-RA	14
AGAP006482-RA	14
AGAP006489-RA	14
AGAP006497-RA	14
AGAP006501-RA	14
AGAP006502-RA	14
AGAP006523-RA	14
AGAP006527-RA	14
AGAP006591-RA	14
AGAP006627-RA	14
AGAP006635-RA	14
AGAP006658-RA	14
AGAP006661-RA	14
AGAP006666-RA	14
AGAP006668-RA	14
AGAP006671-RA	14
AGAP006717-RA	14
AGAP006734-RA	14
AGAP006775-RA	14
AGAP006786-RA	14
AGAP006797-RA	14
AGAP006823-RA	14
AGAP006828-RA	14
AGAP006868-RB	14
AGAP006882-RA	14
AGAP006891-RA	14
AGAP006903-RA	14
AGAP006930-RA	14
AGAP006938-RA	14
AGAP006955-RA	14
AGAP006974-RA	14
AGAP006976-RA	14
AGAP006986-RA	14
AGAP007004-RA	14
AGAP007042-RA	14

AGAP007056-RA	14
AGAP007060-RA	14
AGAP007081-RA	14
AGAP007094-RA	14
AGAP007100-RA	14
AGAP007105-RA	14
AGAP007112-RA	14
AGAP007124-RA	14
AGAP007136-RA	14
AGAP007155-RA	14
AGAP007156-RA	14
AGAP007178-RA	14
AGAP007205-RA	14
AGAP007239-RA	14
AGAP007266-RA	14
AGAP007269-RA	14
AGAP007270-RA	14
AGAP007272-RA	14
AGAP007300-RA	14
AGAP007304-RA	14
AGAP007305-RA	14
AGAP007322-RA	14
AGAP007341-RA	14
AGAP007353-RA	14
AGAP007359-RA	14
AGAP007442-RA	14
AGAP007443-RA	14
AGAP007483-RA	14
AGAP007488-RA	14
AGAP007498-RA	14
AGAP007510-RA	14
AGAP007533-RA	14
AGAP007557-RA	14
AGAP007564-RA	14
AGAP007566-RA	14
AGAP007615-RA	14
AGAP007616-RA	14
AGAP007622-RA	14
AGAP007641-RA	14
AGAP007677-RA	14
AGAP007692-RA	14
AGAP007762-RA	14
AGAP007767-RA	14
AGAP007800-RA	14
AGAP007832-RA	14
AGAP007905-RA	14
AGAP007909-RA	14
AGAP007929-RA	14
AGAP007962-RA	14
AGAP008010-RA	14
AGAP008015-RA	14
AGAP008023-RA	14
AGAP008055-RA	14
AGAP008067-RA	14

AGAP008093-RA	14
AGAP008114-RA	14
AGAP008130-RA	14
AGAP008133-RA	14
AGAP008155-RA	14
AGAP008178-RA	14
AGAP008179-RA	14
AGAP008201-RA	14
AGAP008205-RA	14
AGAP008261-RA	14
AGAP008262-RA	14
AGAP008302-RA	14
AGAP008326-RA	14
AGAP008378-RA	14
AGAP008398-RA	14
AGAP008434-RA	14
AGAP008450-RA	14
AGAP008525-RA	14
AGAP008638-RA	14
AGAP008682-RA	14
AGAP008706-RA	14
AGAP008723-RA	14
AGAP008725-RA	14
AGAP008752-RA	14
AGAP008759-RA	14
AGAP008783-RA	14
AGAP008843-RA	14
AGAP008850-RA	14
AGAP008861-RA	14
AGAP008933-RA	14
AGAP008936-RA	14
AGAP008951-RA	14
AGAP008953-RA	14
AGAP009008-RA	14
AGAP009009-RA	14
AGAP009017-RA	14
AGAP009028-RA	14
AGAP009048-RA	14
AGAP009060-RA	14
AGAP009063-RA	14
AGAP009078-RA	14
AGAP009125-RA	14
AGAP009131-RA	14
AGAP009136-RA	14
AGAP009138-RA	14
AGAP009162-RA	14
AGAP009198-RA	14
AGAP009227-RA	14
AGAP009232-RA	14
AGAP009233-RA	14
AGAP009256-RC	14
AGAP009257-RA	14
AGAP009275-RA	14
AGAP009281-RA	14

AGAP009292-RA	14
AGAP009318-RA	14
AGAP009338-RA	14
AGAP009379-RA	14
AGAP009407-RA	14
AGAP009410-RA	14
AGAP009513-RA	14
AGAP009514-RA	14
AGAP009590-RA	14
AGAP009593-RA	14
AGAP009620-RA	14
AGAP009640-RA	14
AGAP009680-RA	14
AGAP009684-RA	14
AGAP009686-RA	14
AGAP009690-RA	14
AGAP009692-RA	14
AGAP009730-RA	14
AGAP009731-RA	14
AGAP009758-RA	14
AGAP009759-RA	14
AGAP009763-RA	14
AGAP009773-RB	14
AGAP009775-RA	14
AGAP009779-RA	14
AGAP009793-RA	14
AGAP009868-RA	14
AGAP009877-RA	14
AGAP009933-RA	14
AGAP009968-RA	14
AGAP009974-RA	14
AGAP010013-RA	14
AGAP010027-RA	14
AGAP010089-RA	14
AGAP010146-RA	14
AGAP010165-RA	14
AGAP010170-RA	14
AGAP010195-RA	14
AGAP010272-RA	14
AGAP010347-RB	14
AGAP010474-RA	14
AGAP010571-RA	14
AGAP010653-RA	14
AGAP010734-RA	14
AGAP010777-RA	14
AGAP010785-RA	14
AGAP010820-RA	14
AGAP010842-RA	14
AGAP010879-RA	14
AGAP010919-RA	14
AGAP010940-RA	14
AGAP010966-RA	14
AGAP010988-RA	14
AGAP011025-RA	14

AGAP011031-RA	14
AGAP011033-RA	14
AGAP011096-RA	14
AGAP011117-RA	14
AGAP011144-RA	14
AGAP011187-RA	14
AGAP011196-RA	14
AGAP011212-RA	14
AGAP011215-RA	14
AGAP011216-RA	14
AGAP011220-RA	14
AGAP011234-RA	14
AGAP011303-RA	14
AGAP011321-RA	14
AGAP011348-RA	14
AGAP011361-RA	14
AGAP011366-RA	14
AGAP011370-RA	14
AGAP011371-RA	14
AGAP011398-RA	14
AGAP011420-RA	14
AGAP011440-RA	14
AGAP011467-RA	14
AGAP011506-RA	14
AGAP011507-RA	14
AGAP011511-RA	14
AGAP011513-RA	14
AGAP011530-RA	14
AGAP011540-RA	14
AGAP011553-RA	14
AGAP011557-RA	14
AGAP011605-RA	14
AGAP011625-RA	14
AGAP011646-RA	14
AGAP011665-RA	14
AGAP011680-RA	14
AGAP011704-RA	14
AGAP011707-RA	14
AGAP011721-RA	14
AGAP011731-RA	14
AGAP011752-RA	14
AGAP011795-RA	14
AGAP011809-RA	14
AGAP011899-RA	14
AGAP011905-RA	14
AGAP011910-RA	14
AGAP011912-RA	14
AGAP011919-RA	14
AGAP011935-RA	14
AGAP011947-RA	14
AGAP011951-RA	14
AGAP011968-RA	14
AGAP011987-RA	14
AGAP011991-RA	14

AGAP011992-RA	14
AGAP012047-RA	14
AGAP012109-RA	14
AGAP012120-RA	14
AGAP012125-RA	14
AGAP012130-RA	14
AGAP012155-RA	14
AGAP012164-RA	14
AGAP012225-RA	14
AGAP012241-RA	14
AGAP012244-RA	14
AGAP012257-RA	14
AGAP012318-RA	14
AGAP012326-RA	14
AGAP012353-RA	14
AGAP012378-RA	14
AGAP012387-RA	14
AGAP012462-RA	14
AGAP012573-RA	14
AGAP012578-RA	14
AGAP012665-RB	14
AGAP012702-RA	14
AGAP012806-RA	14
AGAP012856-RA	14
AGAP012960-RA	14
AGAP012973-RA	14
AGAP012988-RA	14
AGAP012992-RA	14
AGAP013007-RA	14
AGAP013010-RA	14
AGAP013019-RA	14
AGAP013033-RA	14
AGAP013042-RA	14
AGAP013049-RA	14
AGAP013090-RA	14
AGAP013114-RA	14
AGAP013115-RA	14
AGAP013158-RA	14
AGAP013197-RA	14
AGAP013200-RA	14
AGAP013209-RA	14
AGAP013215-RA	14
AGAP013216-RA	14
AGAP013230-RA	14
AGAP013240-RA	14
AGAP013251-RA	14
AGAP013256-RA	14
AGAP013263-RA	14
AGAP013270-RA	14
AGAP013272-RA	14
AGAP013279-RA	14
AGAP013313-RA	14
AGAP013340-RA	14
AGAP013349-RA	14

AGAP013354-RA	14
AGAP013373-RA	14
AGAP013374-RA	14
AGAP013410-RA	14
AGAP013425-RA	14
AGAP013426-RA	14
AGAP013432-RA	14
AGAP013464-RA	14
AGAP013471-RA	14
AGAP013476-RA	14
AGAP013486-RA	14
AGAP013490-RA	14
AGAP013492-RA	14
AGAP013493-RA	14
AGAP013513-RA	14
AGAP013523-RA	14
AGAP013525-RA	14
AGAP013715-RA	14
AGAP013728-RA	14
AGAP013737-RA	14
AGAP027993-RA	14
AGAP028002-RA	14
AGAP028030-RA	14
AGAP028031-RA	14
AGAP028041-RA	14
AGAP028086-RA	14
AGAP028147-RA	14
AGAP028161-RA	14
AGAP028170-RA	14
AGAP028199-RA	14
AGAP028228-RA	14
AGAP028396-RA	14
AGAP028399-RA	14
AGAP028400-RA	14
AGAP028414-RA	14
AGAP028416-RA	14
AGAP028419-RA	14
AGAP028447-RA	14
AGAP028454-RA	14
AGAP028473-RA	14
AGAP028484-RA	14
AGAP028529-RA	14
AGAP028554-RA	14
AGAP028583-RA	14
AGAP028591-RA	14
AGAP028595-RA	14
AGAP028607-RA	14
AGAP028669-RB	14
AGAP028679-RA	14
AGAP028749-RA	14
AGAP007598-RA	14
AGAP006631-RA	14
AGAP013745-RA	14
AGAP001493-RA	14

AGAP002905-RA	14
AGAP008358-RA	14
AGAP010078-RA	14
AGAP010799-RA	14
AGAP011093-RA	14
AGAP013468-RA	14
AGAP000128-RA	14
AGAP000139-RA	14
AGAP000347-RA	14
AGAP002108-RA	14
AGAP007289-RB	14
AGAP008836-RA	14
AGAP028521-RB	14
AGAP001250-RA	14
AGAP000091-RA	14
AGAP028189-RA	14
AGAP000239-RA	14
AGAP010008-RA	14
AGAP005470-RA	14
AGAP004791-RA	14
AGAP000177-RA	14
AGAP011167-RA	14
AGAP004851-RA	14
AGAP006118-RA	14
AGAP007794-RA	14
AGAP000067-RA	14
AGAP001568-RA	14
AGAP002391-RA	14
AGAP009521-RA	14
AGAP000637-RA	14
AGAP007980-RA	14
AGAP009342-RA	14
AGAP013101-RA	15
AGAP028605-RA	15
AGAP028639-RA	15
AGAP000232-RA	15
AGAP000336-RA	15
AGAP000564-RA	15
AGAP000614-RA	15
AGAP000762-RA	15
AGAP000833-RB	15
AGAP000844-RA	15
AGAP000893-RA	15
AGAP000968-RA	15
AGAP001404-RA	15
AGAP001550-RA	15
AGAP001615-RA	15
AGAP001624-RA	15
AGAP001634-RA	15
AGAP001669-RA	15
AGAP001817-RA	15
AGAP002007-RA	15
AGAP002145-RA	15
AGAP002184-RA	15

AGAP002217-RA	15
AGAP002222-RA	15
AGAP002244-RA	15
AGAP002279-RA	15
AGAP002331-RA	15
AGAP002579-RA	15
AGAP002613-RA	15
AGAP002739-RA	15
AGAP002797-RB	15
AGAP002886-RB	15
AGAP002981-RA	15
AGAP003481-RA	15
AGAP003544-RA	15
AGAP003570-RA	15
AGAP003793-RA	15
AGAP003831-RA	15
AGAP003930-RA	15
AGAP004016-RA	15
AGAP004032-RA	15
AGAP004202-RA	15
AGAP004549-RC	15
AGAP004804-RA	15
AGAP005027-RA	15
AGAP005273-RA	15
AGAP005681-RA	15
AGAP005765-RA	15
AGAP005791-RA	15
AGAP005866-RA	15
AGAP005924-RA	15
AGAP005938-RA	15
AGAP006136-RA	15
AGAP006183-RA	15
AGAP006203-RA	15
AGAP006362-RA	15
AGAP006417-RA	15
AGAP006542-RA	15
AGAP006746-RA	15
AGAP006772-RA	15
AGAP006779-RA	15
AGAP007021-RA	15
AGAP007075-RA	15
AGAP007137-RB	15
AGAP007284-RA	15
AGAP007326-RB	15
AGAP007357-RA	15
AGAP007497-RA	15
AGAP007591-RA	15
AGAP007594-RA	15
AGAP007696-RA	15
AGAP007789-RB	15
AGAP007903-RA	15
AGAP007914-RA	15
AGAP007990-RA	15
AGAP008077-RB	15

AGAP008193-RA	15
AGAP008208-RA	15
AGAP008400-RA	15
AGAP008440-RA	15
AGAP008472-RA	15
AGAP008489-RA	15
AGAP008537-RB	15
AGAP008600-RA	15
AGAP008678-RA	15
AGAP008760-RA	15
AGAP008839-RA	15
AGAP008846-RA	15
AGAP009314-RA	15
AGAP009366-RA	15
AGAP009485-RA	15
AGAP009531-RA	15
AGAP009617-RA	15
AGAP009879-RA	15
AGAP010067-RA	15
AGAP010149-RA	15
AGAP010260-RA	15
AGAP010463-RA	15
AGAP011028-RA	15
AGAP011137-RA	15
AGAP011281-RA	15
AGAP011414-RA	15
AGAP011459-RA	15
AGAP011489-RA	15
AGAP011518-RA	15
AGAP011523-RA	15
AGAP011675-RA	15
AGAP011880-RA	15
AGAP011911-RA	15
AGAP012059-RA	15
AGAP012316-RA	15
AGAP012365-RA	15
AGAP012543-RA	15
AGAP012954-RA	15
AGAP013009-RA	15
AGAP013171-RA	15
AGAP013214-RA	15
AGAP028135-RA	15
AGAP028460-RA	15
AGAP028495-RA	15
AGAP028500-RA	15
AGAP028530-RA	15
AGAP028653-RA	15
AGAP000014-RA	15
AGAP000016-RA	15
AGAP000018-RA	15
AGAP000047-RA	15
AGAP000104-RA	15
AGAP000137-RA	15
AGAP000156-RA	15

AGAP000179-RA	15
AGAP000181-RA	15
AGAP000204-RA	15
AGAP000246-RA	15
AGAP000338-RA	15
AGAP000356-RA	15
AGAP000509-RA	15
AGAP000740-RA	15
AGAP000945-RA	15
AGAP000970-RA	15
AGAP001185-RA	15
AGAP001259-RA	15
AGAP001390-RA	15
AGAP001542-RA	15
AGAP001594-RA	15
AGAP001661-RA	15
AGAP001685-RA	15
AGAP001708-RA	15
AGAP001934-RA	15
AGAP001985-RA	15
AGAP002037-RA	15
AGAP002072-RA	15
AGAP002220-RA	15
AGAP002228-RA	15
AGAP002280-RA	15
AGAP002598-RA	15
AGAP002674-RA	15
AGAP003096-RA	15
AGAP003189-RA	15
AGAP003334-RA	15
AGAP003504-RA	15
AGAP003513-RA	15
AGAP003604-RA	15
AGAP003627-RA	15
AGAP003660-RA	15
AGAP004124-RA	15
AGAP004413-RA	15
AGAP004518-RA	15
AGAP004714-RA	15
AGAP005038-RA	15
AGAP005168-RA	15
AGAP005534-RA	15
AGAP005633-RA	15
AGAP005781-RA	15
AGAP005789-RA	15
AGAP005914-RA	15
AGAP006271-RA	15
AGAP006415-RA	15
AGAP006483-RA	15
AGAP006531-RA	15
AGAP006829-RA	15
AGAP006873-RA	15
AGAP006892-RA	15
AGAP006898-RA	15

AGAP007303-RA	15
AGAP007518-RA	15
AGAP007607-RA	15
AGAP007687-RA	15
AGAP008129-RA	15
AGAP008363-RA	15
AGAP008751-RA	15
AGAP008855-RA	15
AGAP009518-RA	15
AGAP010259-RA	15
AGAP010431-RA	15
AGAP010784-RA	15
AGAP011227-RA	15
AGAP011457-RA	15
AGAP011462-RA	15
AGAP011670-RA	15
AGAP011782-RA	15
AGAP012239-RA	15
AGAP012344-RA	15
AGAP013416-RA	15
AGAP013514-RA	15
AGAP013722-RA	15
AGAP028420-RA	15
AGAP028523-RA	15
AGAP000218-RA	15
AGAP000258-RA	15
AGAP000268-RA	15
AGAP000283-RA	15
AGAP000291-RA	15
AGAP000316-RA	15
AGAP000366-RA	15
AGAP000433-RB	15
AGAP000594-RA	15
AGAP000678-RA	15
AGAP000745-RA	15
AGAP000750-RA	15
AGAP000778-RA	15
AGAP000820-RA	15
AGAP000899-RA	15
AGAP001410-RA	15
AGAP001637-RA	15
AGAP001766-RA	15
AGAP001806-RA	15
AGAP001984-RA	15
AGAP001999-RB	15
AGAP002023-RA	15
AGAP002370-RA	15
AGAP002376-RA	15
AGAP003204-RA	15
AGAP003861-RA	15
AGAP004018-RB	15
AGAP004059-RA	15
AGAP004267-RA	15
AGAP004275-RA	15

AGAP004452-RA	15
AGAP004726-RA	15
AGAP005302-RA	15
AGAP005388-RA	15
AGAP006175-RA	15
AGAP006253-RA	15
AGAP006705-RA	15
AGAP007992-RA	15
AGAP008056-RA	15
AGAP008152-RA	15
AGAP008271-RA	15
AGAP008280-RA	15
AGAP008449-RA	15
AGAP008796-RA	15
AGAP009220-RA	15
AGAP009607-RA	15
AGAP011169-RA	15
AGAP028513-RA	15
AGAP000294-RA	15
AGAP000309-RA	15
AGAP000793-RA	15
AGAP000825-RA	15
AGAP000929-RA	15
AGAP000946-RA	15
AGAP001638-RA	15
AGAP001800-RA	15
AGAP002089-RA	15
AGAP002235-RA	15
AGAP002398-RA	15
AGAP002566-RB	15
AGAP003285-RA	15
AGAP003977-RA	15
AGAP004272-RA	15
AGAP005166-RB	15
AGAP005435-RA	15
AGAP005568-RA	15
AGAP005717-RA	15
AGAP005774-RA	15
AGAP005946-RA	15
AGAP006134-RA	15
AGAP006140-RA	15
AGAP006256-RA	15
AGAP007887-RA	15
AGAP007933-RA	15
AGAP008183-RA	15
AGAP008650-RA	15
AGAP009260-RA	15
AGAP011069-RA	15
AGAP013518-RA	15
AGAP000303-RA	15
AGAP000422-RA	15
AGAP002260-RA	15
AGAP008335-RA	15
AGAP011889-RA	15

AGAP004322-RA	15
AGAP002381-RA	15
AGAP003068-RA	15
AGAP005396-RA	15
AGAP011180-RA	15
AGAP012503-RA	15
AGAP003992-RA	15
AGAP004170-RA	15
AGAP000343-RA	15
AGAP005982-RA	15
AGAP008385-RA	15
AGAP010956-RA	15
AGAP011775-RA	15
AGAP001035-RA	15
AGAP001375-RA	15
AGAP010562-RA	15
AGAP000061-RA	15
AGAP002830-RA	15
AGAP004086-RA	15
AGAP000803-RA	15
AGAP000695-RA	15
AGAP001546-RA	15
AGAP007551-RA	15
AGAP012388-RA	15
AGAP007490-RA	15
AGAP005957-RA	15
AGAP008263-RA	15
AGAP009262-RA	15
AGAP003891-RA	15
AGAP005993-RA	15
AGAP011817-RA	15
AGAP001566-RB	15
AGAP001795-RA	15
AGAP002055-RA	15
AGAP000620-RA	15
AGAP006216-RA	15
AGAP010441-RA	15
AGAP028635-RA	15
AGAP000682-RA	15
AGAP003308-RB	15
AGAP006129	TF.5(MAGs)
AGAP012546	TF.5(MAGs)
AGAP001462	TF.5(MAGs)
AGAP002095	TF.5(MAGs)
AGAP008551	TF.5(MAGs)
AGAP009069	TF.5(MAGs)
AGAP006534	TF.5(MAGs)
AGAP009986	TF.5(MAGs)
AGAP002920	TF.5(MAGs)
AGAP010359	TF.8(Lineage)
AGAP011134	TF.8(Lineage)
AGAP000061	TF.8(Lineage)
AGAP010358	TF.8(Lineage)
AGAP009699	TF.8(Lineage)

AGAP008232	TF.8(Lineage)
AGAP005804	TF.15(Testes)
AGAP007539	TF.15(Testes)
AGAP002236	TF.15(Testes)
AGAP005362	TF.15(Testes)
AGAP009889	TF.15(Testes)
AGAP007978	TF.15(Testes)
AGAP006736	TF.15(Testes)
AGAP008845	TF.15(Testes)

AGAP000044	Testes Highly variable
AGAP000165	Testes Highly variable
AGAP000361	Testes Highly variable
AGAP000426	Testes Highly variable
AGAP000496	Testes Highly variable
AGAP000541	Testes Highly variable
AGAP000604	Testes Highly variable
AGAP000624	Testes Highly variable
AGAP000627	Testes Highly variable
AGAP000654	Testes Highly variable
AGAP000655	Testes Highly variable
AGAP000669	Testes Highly variable
AGAP000692	Testes Highly variable
AGAP000694	Testes Highly variable
AGAP000733	Testes Highly variable
AGAP000851	Testes Highly variable
AGAP000883	Testes Highly variable
AGAP000954	Testes Highly variable
AGAP000969	Testes Highly variable
AGAP001023	Testes Highly variable
AGAP001025	Testes Highly variable
AGAP001065	Testes Highly variable
AGAP001165	Testes Highly variable
AGAP001368	Testes Highly variable
AGAP001387	Testes Highly variable
AGAP001416	Testes Highly variable
AGAP001417	Testes Highly variable
AGAP001424	Testes Highly variable
AGAP001446	Testes Highly variable
AGAP001502	Testes Highly variable
AGAP001620	Testes Highly variable
AGAP001680	Testes Highly variable
AGAP001706	Testes Highly variable
AGAP001711	Testes Highly variable
AGAP001728	Testes Highly variable
AGAP001735	Testes Highly variable
AGAP001767	Testes Highly variable
AGAP001813	Testes Highly variable
AGAP001903	Testes Highly variable
AGAP001914	Testes Highly variable
AGAP001952	Testes Highly variable
AGAP002043	Testes Highly variable
AGAP002059	Testes Highly variable
AGAP002076	Testes Highly variable

AGAP002095	Testes	Highly variable
AGAP002098	Testes	Highly variable
AGAP002122	Testes	Highly variable
AGAP002131	Testes	Highly variable
AGAP002216	Testes	Highly variable
AGAP002245	Testes	Highly variable
AGAP002248	Testes	Highly variable
AGAP002282	Testes	Highly variable
AGAP002301	Testes	Highly variable
AGAP002339	Testes	Highly variable
AGAP002353	Testes	Highly variable
AGAP002376	Testes	Highly variable
AGAP002464	Testes	Highly variable
AGAP002467	Testes	Highly variable
AGAP002525	Testes	Highly variable
AGAP002564	Testes	Highly variable
AGAP002609	Testes	Highly variable
AGAP002613	Testes	Highly variable
AGAP002625	Testes	Highly variable
AGAP002686	Testes	Highly variable
AGAP002693	Testes	Highly variable
AGAP002754	Testes	Highly variable
AGAP002880	Testes	Highly variable
AGAP002996	Testes	Highly variable
AGAP003090	Testes	Highly variable
AGAP003112	Testes	Highly variable
AGAP003116	Testes	Highly variable
AGAP003164	Testes	Highly variable
AGAP003184	Testes	Highly variable
AGAP003212	Testes	Highly variable
AGAP003228	Testes	Highly variable
AGAP003275	Testes	Highly variable
AGAP003277	Testes	Highly variable
AGAP003336	Testes	Highly variable
AGAP003411	Testes	Highly variable
AGAP003460	Testes	Highly variable
AGAP003462	Testes	Highly variable
AGAP003475	Testes	Highly variable
AGAP003477	Testes	Highly variable
AGAP003517	Testes	Highly variable
AGAP003592	Testes	Highly variable
AGAP003598	Testes	Highly variable
AGAP003768	Testes	Highly variable
AGAP003786	Testes	Highly variable
AGAP003810	Testes	Highly variable
AGAP003879	Testes	Highly variable
AGAP003919	Testes	Highly variable
AGAP004012	Testes	Highly variable
AGAP004030	Testes	Highly variable
AGAP004064	Testes	Highly variable
AGAP004110	Testes	Highly variable
AGAP004216	Testes	Highly variable
AGAP004221	Testes	Highly variable
AGAP004235	Testes	Highly variable

AGAP004238	Testes	Highly variable
AGAP004245	Testes	Highly variable
AGAP004294	Testes	Highly variable
AGAP004340	Testes	Highly variable
AGAP004459	Testes	Highly variable
AGAP004462	Testes	Highly variable
AGAP004616	Testes	Highly variable
AGAP004626	Testes	Highly variable
AGAP004657	Testes	Highly variable
AGAP004711	Testes	Highly variable
AGAP004819	Testes	Highly variable
AGAP004846	Testes	Highly variable
AGAP004904	Testes	Highly variable
AGAP004911	Testes	Highly variable
AGAP004919	Testes	Highly variable
AGAP004964	Testes	Highly variable
AGAP004987	Testes	Highly variable
AGAP004988	Testes	Highly variable
AGAP005052	Testes	Highly variable
AGAP005081	Testes	Highly variable
AGAP005180	Testes	Highly variable
AGAP005422	Testes	Highly variable
AGAP005467	Testes	Highly variable
AGAP005478	Testes	Highly variable
AGAP005503	Testes	Highly variable
AGAP005600	Testes	Highly variable
AGAP005685	Testes	Highly variable
AGAP005775	Testes	Highly variable
AGAP005844	Testes	Highly variable
AGAP005850	Testes	Highly variable
AGAP005887	Testes	Highly variable
AGAP005890	Testes	Highly variable
AGAP005919	Testes	Highly variable
AGAP005947	Testes	Highly variable
AGAP005972	Testes	Highly variable
AGAP006036	Testes	Highly variable
AGAP006130	Testes	Highly variable
AGAP006348	Testes	Highly variable
AGAP006369	Testes	Highly variable
AGAP006391	Testes	Highly variable
AGAP006406	Testes	Highly variable
AGAP006409	Testes	Highly variable
AGAP006482	Testes	Highly variable
AGAP006603	Testes	Highly variable
AGAP006662	Testes	Highly variable
AGAP006706	Testes	Highly variable
AGAP006756	Testes	Highly variable
AGAP006766	Testes	Highly variable
AGAP006924	Testes	Highly variable
AGAP007144	Testes	Highly variable
AGAP007202	Testes	Highly variable
AGAP007206	Testes	Highly variable
AGAP007213	Testes	Highly variable
AGAP007240	Testes	Highly variable

AGAP007297	Testes	Highly variable
AGAP007373	Testes	Highly variable
AGAP007374	Testes	Highly variable
AGAP007383	Testes	Highly variable
AGAP007393	Testes	Highly variable
AGAP007458	Testes	Highly variable
AGAP007459	Testes	Highly variable
AGAP007473	Testes	Highly variable
AGAP007668	Testes	Highly variable
AGAP007699	Testes	Highly variable
AGAP007732	Testes	Highly variable
AGAP007749	Testes	Highly variable
AGAP007800	Testes	Highly variable
AGAP007806	Testes	Highly variable
AGAP007853	Testes	Highly variable
AGAP007893	Testes	Highly variable
AGAP007917	Testes	Highly variable
AGAP007966	Testes	Highly variable
AGAP008049	Testes	Highly variable
AGAP008113	Testes	Highly variable
AGAP008163	Testes	Highly variable
AGAP008213	Testes	Highly variable
AGAP008219	Testes	Highly variable
AGAP008287	Testes	Highly variable
AGAP008329	Testes	Highly variable
AGAP008402	Testes	Highly variable
AGAP008405	Testes	Highly variable
AGAP008432	Testes	Highly variable
AGAP008530	Testes	Highly variable
AGAP008539	Testes	Highly variable
AGAP008540	Testes	Highly variable
AGAP008635	Testes	Highly variable
AGAP008686	Testes	Highly variable
AGAP008727	Testes	Highly variable
AGAP008905	Testes	Highly variable
AGAP008921	Testes	Highly variable
AGAP008970	Testes	Highly variable
AGAP009019	Testes	Highly variable
AGAP009032	Testes	Highly variable
AGAP009080	Testes	Highly variable
AGAP009084	Testes	Highly variable
AGAP009260	Testes	Highly variable
AGAP009263	Testes	Highly variable
AGAP009314	Testes	Highly variable
AGAP009325	Testes	Highly variable
AGAP009431	Testes	Highly variable
AGAP009441	Testes	Highly variable
AGAP009446	Testes	Highly variable
AGAP009447	Testes	Highly variable
AGAP009459	Testes	Highly variable
AGAP009491	Testes	Highly variable
AGAP009593	Testes	Highly variable
AGAP009616	Testes	Highly variable
AGAP009629	Testes	Highly variable

AGAP009658	Testes Highly variable
AGAP009737	Testes Highly variable
AGAP009750	Testes Highly variable
AGAP009771	Testes Highly variable
AGAP009984	Testes Highly variable
AGAP010005	Testes Highly variable
AGAP010130	Testes Highly variable
AGAP010163	Testes Highly variable
AGAP010188	Testes Highly variable
AGAP010199	Testes Highly variable
AGAP010212	Testes Highly variable
AGAP010251	Testes Highly variable
AGAP010253	Testes Highly variable
AGAP010267	Testes Highly variable
AGAP010586	Testes Highly variable
AGAP010588	Testes Highly variable
AGAP010871	Testes Highly variable
AGAP010919	Testes Highly variable
AGAP010929	Testes Highly variable
AGAP010954	Testes Highly variable
AGAP011098	Testes Highly variable
AGAP011101	Testes Highly variable
AGAP011114	Testes Highly variable
AGAP011232	Testes Highly variable
AGAP011324	Testes Highly variable
AGAP011329	Testes Highly variable
AGAP011338	Testes Highly variable
AGAP011378	Testes Highly variable
AGAP011423	Testes Highly variable
AGAP011424	Testes Highly variable
AGAP011439	Testes Highly variable
AGAP011472	Testes Highly variable
AGAP011497	Testes Highly variable
AGAP011516	Testes Highly variable
AGAP011580	Testes Highly variable
AGAP011616	Testes Highly variable
AGAP011687	Testes Highly variable
AGAP011803	Testes Highly variable
AGAP011829	Testes Highly variable
AGAP011833	Testes Highly variable
AGAP011845	Testes Highly variable
AGAP011895	Testes Highly variable
AGAP011896	Testes Highly variable
AGAP011945	Testes Highly variable
AGAP011994	Testes Highly variable
AGAP012029	Testes Highly variable
AGAP012045	Testes Highly variable
AGAP012082	Testes Highly variable
AGAP012135	Testes Highly variable
AGAP012145	Testes Highly variable
AGAP012184	Testes Highly variable
AGAP012291	Testes Highly variable
AGAP012308	Testes Highly variable
AGAP012407	Testes Highly variable

AGAP012636	Testes Highly variable
AGAP012828	Testes Highly variable
AGAP012973	Testes Highly variable
AGAP013168	Testes Highly variable
AGAP013212	Testes Highly variable
AGAP013338	Testes Highly variable
AGAP013462	Testes Highly variable
AGAP013731	Testes Highly variable
AGAP013745	Testes Highly variable
AGAP013750	Testes Highly variable
AGAP028100	Testes Highly variable
AGAP028447	Testes Highly variable
AGAP028460	Testes Highly variable
AGAP028485	Testes Highly variable
AGAP028489	Testes Highly variable
AGAP028529	Testes Highly variable
AGAP028599	Testes Highly variable
AGAP028601	Testes Highly variable
AGAP028693	Testes Highly variable
AGAP028740	Testes Highly variable
AGAP009360	Testes Highly variable
AGAP028039	Testes Highly variable
AGAP009367	Testes Highly variable
AGAP009369	Testes Highly variable
AGAP009368	Testes Highly variable
AGAP012706	Testes Highly variable
AGAP009370	Testes Highly variable
AGAP005791	MAGs Highly variable
AGAP028693	MAGs Highly variable
AGAP007393	MAGs Highly variable
AGAP008276	MAGs Highly variable
AGAP010251	MAGs Highly variable
AGAP006036	MAGs Highly variable
AGAP001424	MAGs Highly variable
AGAP004461	MAGs Highly variable
AGAP012082	MAGs Highly variable
AGAP028740	MAGs Highly variable
AGAP008141	MAGs Highly variable
AGAP008329	MAGs Highly variable
AGAP011829	MAGs Highly variable
AGAP002376	MAGs Highly variable
AGAP012706	MAGs Highly variable
AGAP003592	MAGs Highly variable
AGAP000541	MAGs Highly variable
AGAP008635	MAGs Highly variable
AGAP003184	MAGs Highly variable
AGAP002076	MAGs Highly variable
AGAP004381	MAGs Highly variable
AGAP008727	MAGs Highly variable
AGAP009369	MAGs Highly variable
AGAP010253	MAGs Highly variable
AGAP001711	MAGs Highly variable
AGAP012926	MAGs Highly variable
AGAP005947	MAGs Highly variable

AGAP010929	MAGs Highly variable
AGAP008686	MAGs Highly variable
AGAP009370	MAGs Highly variable
AGAP002754	MAGs Highly variable
AGAP010163	MAGs Highly variable
AGAP006371	MAGs Highly variable
AGAP001174	MAGs Highly variable
AGAP002131	MAGs Highly variable
AGAP008892	MAGs Highly variable
AGAP006582	MAGs Highly variable
AGAP011423	MAGs Highly variable
AGAP013351	MAGs Highly variable
AGAP005627	MAGs Highly variable
AGAP003879	MAGs Highly variable
AGAP005833	MAGs Highly variable
AGAP003277	MAGs Highly variable
AGAP001813	MAGs Highly variable
AGAP000851	MAGs Highly variable
AGAP006348	MAGs Highly variable
AGAP012407	MAGs Highly variable
AGAP007781	MAGs Highly variable
AGAP004400	MAGs Highly variable
AGAP009737	MAGs Highly variable
AGAP004846	MAGs Highly variable
AGAP006756	MAGs Highly variable
AGAP013731	MAGs Highly variable
AGAP011842	MAGs Highly variable
AGAP000627	MAGs Highly variable
AGAP005081	MAGs Highly variable
AGAP003411	MAGs Highly variable
AGAP010954	MAGs Highly variable
AGAP009446	MAGs Highly variable
AGAP001065	MAGs Highly variable
AGAP009491	MAGs Highly variable
AGAP011497	MAGs Highly variable
AGAP001417	MAGs Highly variable
AGAP004452	MAGs Highly variable
AGAP005459	MAGs Highly variable
AGAP005467	MAGs Highly variable
AGAP002464	MAGs Highly variable
AGAP008353	MAGs Highly variable
AGAP000310	MAGs Highly variable
AGAP002686	MAGs Highly variable
AGAP005790	MAGs Highly variable
AGAP006043	MAGs Highly variable
AGAP004657	MAGs Highly variable
AGAP008049	MAGs Highly variable
AGAP013338	MAGs Highly variable
AGAP008204	MAGs Highly variable
AGAP007459	MAGs Highly variable
AGAP003267	MAGs Highly variable
AGAP006130	MAGs Highly variable
AGAP012135	MAGs Highly variable
AGAP006924	MAGs Highly variable

AGAP007202	MAGs Highly variable
AGAP012587	MAGs Highly variable
AGAP012045	MAGs Highly variable
AGAP006386	MAGs Highly variable
AGAP003475	MAGs Highly variable
AGAP008203	MAGs Highly variable
AGAP006934	MAGs Highly variable
AGAP011845	MAGs Highly variable
AGAP012145	MAGs Highly variable
AGAP004046	MAGs Highly variable
AGAP002609	MAGs Highly variable
AGAP001502	MAGs Highly variable
AGAP007699	MAGs Highly variable
AGAP006970	MAGs Highly variable
AGAP008530	MAGs Highly variable
AGAP000177	MAGs Highly variable
AGAP000181	MAGs Highly variable
AGAP009373	MAGs Highly variable
AGAP010188	MAGs Highly variable
AGAP000496	MAGs Highly variable
AGAP005844	MAGs Highly variable
AGAP009367	MAGs Highly variable
AGAP012291	MAGs Highly variable
AGAP010606	MAGs Highly variable
AGAP004030	MAGs Highly variable
AGAP001447	MAGs Highly variable
AGAP004987	MAGs Highly variable
AGAP009263	MAGs Highly variable
AGAP001387	MAGs Highly variable
AGAP010384	MAGs Highly variable
AGAP000733	MAGs Highly variable
AGAP007373	MAGs Highly variable
AGAP007966	MAGs Highly variable
AGAP005289	MAGs Highly variable
AGAP004626	MAGs Highly variable
AGAP001314	MAGs Highly variable
AGAP003786	MAGs Highly variable
AGAP011945	MAGs Highly variable
AGAP002525	MAGs Highly variable
AGAP009182	MAGs Highly variable
AGAP004141	MAGs Highly variable
AGAP007491	MAGs Highly variable
AGAP008432	MAGs Highly variable
AGAP009319	MAGs Highly variable
AGAP010078	MAGs Highly variable
AGAP001712	MAGs Highly variable
AGAP004064	MAGs Highly variable
AGAP000426	MAGs Highly variable
AGAP004110	MAGs Highly variable
AGAP003795	MAGs Highly variable
AGAP008113	MAGs Highly variable
AGAP010894	MAGs Highly variable
AGAP002467	MAGs Highly variable
AGAP009934	MAGs Highly variable

AGAP003336	MAGs Highly variable
AGAP005890	MAGs Highly variable
AGAP004071	MAGs Highly variable
AGAP008163	MAGs Highly variable
AGAP000433	MAGs Highly variable
AGAP005413	MAGs Highly variable
AGAP002407	MAGs Highly variable
AGAP007893	MAGs Highly variable
AGAP000624	MAGs Highly variable
AGAP011803	MAGs Highly variable
AGAP001804	MAGs Highly variable
AGAP007383	MAGs Highly variable
AGAP010613	MAGs Highly variable
AGAP005685	MAGs Highly variable
AGAP010871	MAGs Highly variable
AGAP028031	MAGs Highly variable
AGAP012290	MAGs Highly variable
AGAP002920	MAGs Highly variable
AGAP001903	MAGs Highly variable
AGAP008449	MAGs Highly variable
AGAP011775	MAGs Highly variable
AGAP004668	MAGs Highly variable
AGAP004904	MAGs Highly variable
AGAP001983	MAGs Highly variable
AGAP010477	MAGs Highly variable
AGAP002996	MAGs Highly variable
AGAP004294	MAGs Highly variable
AGAP001764	MAGs Highly variable
AGAP008287	MAGs Highly variable
AGAP028038	MAGs Highly variable
AGAP002921	MAGs Highly variable
AGAP005649	MAGs Highly variable
AGAP012283	MAGs Highly variable
AGAP010130	MAGs Highly variable
AGAP005585	MAGs Highly variable
AGAP001706	MAGs Highly variable
AGAP009049	MAGs Highly variable
AGAP011424	MAGs Highly variable
AGAP003810	MAGs Highly variable
AGAP007806	MAGs Highly variable
AGAP013168	MAGs Highly variable
AGAP011896	MAGs Highly variable
AGAP009967	MAGs Highly variable
AGAP002059	MAGs Highly variable
AGAP010799	MAGs Highly variable
AGAP000843	MAGs Highly variable
AGAP009431	MAGs Highly variable
AGAP008921	MAGs Highly variable
AGAP012463	MAGs Highly variable
AGAP003968	MAGs Highly variable
AGAP002301	MAGs Highly variable
AGAP003275	MAGs Highly variable
AGAP005131	MAGs Highly variable
AGAP001732	MAGs Highly variable

AGAP005129	MAGs Highly variable
AGAP007693	MAGs Highly variable
AGAP009109	MAGs Highly variable
AGAP028100	MAGs Highly variable
AGAP008122	MAGs Highly variable
AGAP005830	MAGs Highly variable
AGAP003212	MAGs Highly variable
AGAP007473	MAGs Highly variable
AGAP004919	MAGs Highly variable
AGAP007668	MAGs Highly variable
AGAP011635	MAGs Highly variable
AGAP008888	MAGs Highly variable
AGAP002904	MAGs Highly variable
AGAP006388	MAGs Highly variable
AGAP001008	MAGs Highly variable
AGAP002543	MAGs Highly variable
AGAP004238	MAGs Highly variable
AGAP013186	MAGs Highly variable
AGAP000321	MAGs Highly variable
AGAP012636	MAGs Highly variable
AGAP005972	MAGs Highly variable
AGAP001546	MAGs Highly variable
AGAP009127	MAGs Highly variable
AGAP004080	MAGs Highly variable
AGAP010405	MAGs Highly variable
AGAP002625	MAGs Highly variable
AGAP008082	MAGs Highly variable
AGAP001760	MAGs Highly variable
AGAP003012	MAGs Highly variable
AGAP006649	MAGs Highly variable
AGAP009635	MAGs Highly variable
AGAP000044	MAGs Highly variable
AGAP008034	MAGs Highly variable
AGAP011329	MAGs Highly variable
AGAP011946	MAGs Highly variable
AGAP009192	MAGs Highly variable
AGAP010785	MAGs Highly variable
AGAP007917	MAGs Highly variable
AGAP006408	MAGs Highly variable
AGAP007297	MAGs Highly variable
AGAP011636	MAGs Highly variable
AGAP001914	MAGs Highly variable
AGAP012521	MAGs Highly variable
AGAP003768	MAGs Highly variable
AGAP011833	MAGs Highly variable
AGAP003486	MAGs Highly variable
