# Supplementary material for: Evolution of gene expression levels in the male reproductive organs of Anopheles mosquitoes
Source: Life Sci Alliance. 2019 Jan 2;2(1):e201800191. doi: 10.26508/lsa.201800191 (PMC6315087; doi:10.26508/lsa.201800191)
Supplement: Supplementary file 4 [file LSA-2018-00191_TableS4.pdf]

ID	Omega	Chromosome/scaffold name	Start	End	Gene description
AGAP005534-RA	1.086	2L	16691245	16692514	
AGAP005685-RA	1.423	2L	18746156	18746953	
AGAP005818-RA	1.047	2L	21831271	21832371	nurim homolog [Source:VB Community Annotation]
AGAP006095-RA	1.455	2L	26615393	26616178	cuticular protein RR-1 family 106 [Source:VB Community Annotation]
AGAP006253-RA	7.178	2L	28620618	28621149	Cysteine-rich venom protein [Source:VB Community Annotation]
AGAP006409-RA	8.694	2L	31569083	31569433	
AGAP006706-RA	1.445	2L	37112415	37113213	
AGAP006830-RA	1.157	2L	39087872	39093776	cuticular protein RR-1 family 58 [Source:VB Community Annotation]
AGAP007313-RA	1.046	2L	45309741	45310625	
AGAP001712-RA	1.061	2R	8859258	8860220	actin related protein 2/3 complex, subunit 3 [Source:VB Community Annotation]
AGAP002553-RA	3.496	2R	22774444	22776503	
AGAP004452-RA	1.321	2R	56391403	56398232	
AGAP008449-RA	5.134	3R	10877642	10878471	cuticular protein CPLCG family (CPLCG5) [Source:VB Community Annotation]
AGAP009372-RA	1.033	3R	31825768	31826272	Acp
AGAP009429-RA	1.372	3R	33439610	33440066	
AGAP009635-RA	1.047	3R	37367914	37368590	Acp
AGAP009879-RA	4.558	3R	44722312	44724555	cuticular protein RR-1 family 81 [Source:VB Community Annotation]
AGAP000072-RA	1.005	X	1120489	1121931	
AGAP000278-RA	1.409	X	5035248	5036100	odorant-binding protein 9 [Source:VB Community Annotation]
AGAP000392-RA	1.373	X	7190193	7191535	synaptobrevin homolog YKT6 [Source:VB Community Annotation]
AGAP000395-RA	1.59	X	7231460	7232678	endoplasmic reticulum protein [Source:VB Community Annotation]
AGAP000433-RB	999	X	7694969	7699060	Ras-related protein Rab-39B [Source:VB Community Annotation]
AGAP000541-RB	999	X	9680497	9683814	40S ribosomal protein S15a [Source:VB Community Annotation]
AGAP000805-RA	1.385	X	14756843	14758303	BTB/POZ domain-containing protein KCTD16 [Source:VB Community Annotation]
